# Genome-wide association study of cognitive function in diverse Hispanics/Latinos: results from the Hispanic Community Health Study/Study of Latinos

**DOI:** 10.1038/s41398-020-00930-2

**Published:** 2020-07-22

**Authors:** Xueqiu Jian, Tamar Sofer, Wassim Tarraf, Jan Bressler, Jessica D. Faul, Wei Zhao, Scott M. Ratliff, Melissa Lamar, Lenore J. Launer, Cathy C. Laurie, Neil Schneiderman, David R. Weir, Clinton B. Wright, Kristine Yaffe, Donglin Zeng, Charles DeCarli, Thomas H. Mosley, Jennifer A. Smith, Hector M. González, Myriam Fornage

**Affiliations:** 1 Brown Foundation Institute of Molecular Medicine, McGovern Medical School, The University of Texas Health Science Center at Houston, Houston, TX USA; 2 Glenn Biggs Institute for Alzheimer’s & Neurodegenerative Diseases, The University of Texas Health Science Center at San Antonio, San Antonio, TX USA; 3 Brigham and Women’s Hospital, Harvard Medical School, Boston, MA USA; 4 Institute of Gerontology and Department of Health Care Sciences, Wayne State University, Detroit, MI USA; 5 Department of Epidemiology, Human Genetics and Environmental Sciences and Human Genetics Center, The University of Texas Health Science Center at Houston School of Public Health, Houston, TX USA; 6 Survey Research Center, Institute for Social Research, University of Michigan, Ann Arbor, MI USA; 7 Department of Epidemiology, University of Michigan School of Public Health, Ann Arbor, MI USA; 8 Department of Behavioral Sciences, Rush Medical College, Chicago, IL USA; 9 Laboratory of Epidemiology and Population Science, National Institute on Aging, Bethesda, MD USA; 10 Department of Biostatistics, University of Washington School of Public Health, Seattle, WA USA; 11 Department of Psychology, University of Miami, Coral Gables, FL USA; 12 Division of Clinical Research, National Institute of Neurological Disorders and Stroke, Bethesda, MD USA; 13 Department of Psychiatry, University of California, San Francisco, San Francisco, CA USA; 14 Department of Biostatistics, Gillings School of Global Public Health, University of North Carolina, Chapel Hill, NC USA; 15 Department of Neurology, School of Medicine and Imaging of Dementia and Aging Laboratory, Center for Neuroscience, University of California, Davis, Sacramento, CA USA; 16 Memory Impairment and Neurodegenerative Dementia (MIND) Center and Department of Medicine, The University of Mississippi Medical Center, Jackson, MS USA; 17 Department of Neurosciences and Shiley-Marcos Alzheimer’s Disease Research Center, University of California, San Diego, La Jolla, CA USA

**Keywords:** Genomics, Biomarkers

## Abstract

Cognitive function such as reasoning, attention, memory, and language is strongly correlated with brain aging. Compared to non-Hispanic whites, Hispanics/Latinos have a higher risk of cognitive impairment and dementia. The genetic determinants of cognitive function have not been widely explored in this diverse and admixed population. We conducted a genome-wide association analysis of cognitive function in up to 7600 middle aged and older Hispanics/Latinos (mean = 55 years) from the Hispanic Community Health Study / Study of Latinos (HCHS/SOL). Four cognitive measures were examined: the Brief Spanish English Verbal Learning Test (B-SEVLT), the Word Fluency Test (WFT), the Digit Symbol Substitution Test (DSST), the Six-Item Screener (SIS). Four novel loci were identified: one for B-SEVLT at 4p14, two for WFT at 3p14.1 and 6p21.32, and one for DSST at 10p13. These loci implicate genes highly expressed in brain and previously connected to neurological diseases (*UBE2K*, *FRMD4B*, the *HLA* gene complex). By applying tissue-specific gene expression prediction models to our genotype data, additional genes highly expressed in brain showed suggestive associations with cognitive measures possibly indicating novel biological mechanisms, including *IFT122* in the hippocampus for SIS, *SNX31* in the basal ganglia for B-SEVLT, *RPS6KB2* in the frontal cortex for WFT, and *CSPG5* in the hypothalamus for DSST. These findings provide new information about the genetic determinants of cognitive function in this unique population. In addition, we derived a measure of general cognitive function based on these cognitive tests and generated genome-wide association summary results, providing a resource to the research community for comparison, replication, and meta-analysis in future genetic studies in Hispanics/Latinos.

## Introduction

Cognitive function refers to a set of cerebral activities such as reasoning, attention, memory, and language, which are supported by specific neuronal networks in the brain. These functions are highly correlated with brain aging. Various cognitive tests were developed to assess different aspects of these functions quickly and non-invasively, and have proven to be valid measures of brain function and the onset of dementia^1–4^. Twin studies suggested that inter-individual variation in cognitive function has a genetic component^5–8^. Several genome-wide association studies (GWAS) have been conducted and identified common variants associated with various measures of cognitive function^9–13^, which aside from the apolipoprotein E (*APOE*) region have yielded a limited number of replicable loci. However, these have been conducted primarily in populations of European ancestry^14^. The Hispanic/Latino population is the largest ethnic/racial minority group in the United States (US), with an estimate of 58.9 million, representing 18.1% of the total US population in 2017^15^. The risk of cognitive impairment and dementia is higher in US ethnic/racial minorities such as Hispanics/Latinos than in non-Hispanic whites^16^. US Hispanic/Latino populations are diverse in many respects, including history, culture, and socio-economic factors. They also vary in cognitive function, which cannot be fully explained by these factors^17^. Genetically, the US Hispanic/Latino population is uniquely admixed, representing Amerindian, African, and European continental ancestries^18^. Results from GWAS in populations of European ancestry may or may not be generalizable to Hispanic/Latino populations. While some GWAS studies of late-onset Alzheimer’s disease (LOAD) have been carried out in Caribbean Hispanics (Dominican and Puerto Rican)^19,20^, to our knowledge, no GWAS of cognitive function has, to date, been reported in a diverse Hispanic/Latino population that includes Cubans, Dominicans, Puerto Ricans, Mexicans, Central Americans, and South Americans.

In this study, we sought to examine genetic associations with cognitive function among diverse middle-aged and older Hispanics/Latinos within the Hispanic Community Health Study/Study of Latinos (HCHS/SOL).

## Materials and methods

### Study sample

The subjects were drawn from the HCHS/SOL, a community-based cohort study with the goal of identifying risk or protective factors for cardiovascular and pulmonary diseases among diverse US Hispanics/Latinos. A total of 16 415 self-identified Hispanic/Latino adults, 18 to 74 years old, were recruited from four US metropolitan areas (Bronx, New York; Chicago, Illinois; Miami, Florida; and San Diego, California) between 2008 and 2011. Various biospecimen and health information about risk/protective factors were collected at the Visit 1 examination^21,22^. The HCHS/SOL study was approved by institutional review boards at participating institutions, including each field center, coordinating center, and the University of Texas Health Science Center at Houston. Written informed consent was obtained from all participants.

### Measures of cognitive function

A subset of the HCHS/SOL sample with middle-aged and older participants (age ≥ 45 years, sample size = 9652) underwent cognitive assessment including the Six-Item Screener (SIS, global mental status), Brief Spanish English Verbal Learning Test (B-SEVLT, verbal learning and memory), Word Fluency Test (WFT, executive and verbal functioning), and Digit Symbol Substitution Test (DSST, psychomotor speed and sustained attention)^17^. We did not exclude patients with dementia or mild cognitive impairment because they were not ascertained at this visit. Each test has been previously described^17^ and is summarized in Supplementary Table 1. Because the distribution of the SIS score is skewed in our sample, we dichotomized this measure, with a score of 0 to 4 indicating low mental status and 5 or 6 indicating normal mental status^17^. We also derived a measure of general cognitive function (G) for each study participants as described by Davies et al.^14^. Specifically, G is defined as the value on the first unrotated principal component (PC1) of the standardized scores for B-SEVLT, WFT, and DSST.

### Genotyping, imputation and quality control

Details of genotyping and imputation were reported elsewhere^23^. In brief, 12,803 participants were successfully genotyped at ~2.4 million variants including single nucleotide variants (SNVs) and insertions/deletions (indels) on an Illumina custom array and passed the standard quality-assurance and quality-control procedures^24^. These genotypes were then pre-phased, followed by imputation with the 1000 Genomes Phase III reference panel, yielding over 50 million imputed variants. For association testing additional quality control was applied based on effective minor allele count (effN), which was defined as *2 × MAF × (1* − *MAF) × sample size × imputation quality*, where MAF is the minor allele frequency of a variant in the sample. A single variant was assessed for association if effN ≥ 30 for quantitative measure (B-SEVLT, WFT, DSST, and G) or effN ≥ 50 in both categories for binary measure (dichotomized SIS).

### Genome-wide association analyses

We performed genetic association analyses with measures of cognitive function using a linear mixed model (LMM). For dichotomized SIS, we used the penalized quasi-likelihood method to approximate the more computationally intensive generalized LMM^25^. In the mixed model for each measure, we included sex, baseline age, recruitment center, sampling weight that corrects for potential bias introduced by the sampling procedure, and the top five principal components that account for population stratification due to ancestry variation, as fixed effects. In addition, we included the “genetic-analysis group”, a categorical variable (Central American, Cuban, Dominican, Mexican, Puerto Rican, and South American) constructed based on self-identified background group and genetic variation (thus more genetically homogeneous within groups), as a fixed effect. For quantitative cognitive measures, we further specified the model to allow for heterogeneous residual variances among ancestry groups defined by the “genetic-analysis group”^18^. Meanwhile, we fitted the mixed model with random effects due to kinship, household, and block group. All analyses were performed with the GENetic EStimation and Inference in Structured samples (GENESIS) Bioconductor package^26^. For each cognitive measure, the threshold of *p* < 5 × 10^−8^ was used to identify genome-wide significant variants.

Because cognitive function and education are correlated both genetically and phenotypically, our primary analyses did not adjust for education so as to maximize our power to identify genetic variants. However, in secondary analyses, education level (<high school, = high school, or >high school) was included as a covariate to assess whether our genetic findings are confounded by education. Genome-wide association analyses were conducted in a total sample of 7606 individuals with available genotype and phenotype data.

### Ancestry-specific allele frequency estimate for top variants and their replication in independent samples

To help select the appropriate population for replication of our top GWAS variants, we first estimated their ancestry-specific allele frequencies using the ASAFE algorithm^27^. Our replication sample included three studies: the Atherosclerosis Risk in Communities (ARIC) study^28^, the Coronary Artery Risk Development in Young Adults (CARDIA) study^29^, and the Health and Retirement Study (HRS)^30^. All three studies contain individuals of European and African ancestries. A proportion of the HRS participants are Hispanic. A detailed description of the replication samples is provided in Supplementary Methods. Measures of cognitive function in each of the replication studies as well as the demographic information are summarized in Supplementary Table 2. Linear (or logistic) regression models were used to test the association of selected variants with measures of cognitive function in the replication samples. Results from each replication sample were then combined by fixed effect meta-analysis.

### Lookup of previously identified GWAS signals and meta-analysis of general cognitive function

In our data, we looked up genome-wide significant variants reported in the recently published largest GWAS of general cognitive function in the population of European ancestry by Davies et al. (*N* > 300,000)^14^, using a Bonferroni-corrected threshold for 148 independent loci identified. Reciprocally, we also examined whether any of our genome-wide significant loci showed evidence of association by Davies et al.^14^. Because our identified variants may, themselves, not be causal but rather may tag (a) potential causal variant(s) and because of differences in linkage disequilibrium (LD) among the populations in the two studies, we performed a look-up on all proxies in LD with our top variants in Europeans (*r*^2^ ≥ 0.6) and we examined all variants within 100 kb of our top variant at each locus.

To identify additional loci for general cognitive function, we meta-analyzed the GWAS results for general cognitive function in the two studies, restricting to over 20 000 variants that showed suggestive association (10^−5^ < *p* < 5 × 10^−8^) in the Davies et al GWAS, using a z-based N-weighted approach.

### Phenotype correlation, heritability and genetic correlation estimate

The correlation between each pair of cognitive measures in our study was estimated using Pearson correlation. We then applied the GREML approach implemented in the GCTA^31^ to estimate variance explained by common variants for each measure of cognitive function, using the kinship coefficients estimated by Conomos et al.^18^. We also used the bivariate GREML method to estimate the genetic correlation between each pair of the cognitive function measures^32^. Compared to LD-based methods, the GREML provides more accurate estimates while requiring smaller sample sizes.

### Fine-mapping GWAS loci using epigenomic annotations

To further prioritize risk variants within a locus, we integrated epigenomic annotations with GWAS summary statistics using a Bayesian framework RiVIERA^33^. We first obtained narrow peaks for the histone ChIP-seq and DNase-seq for 848 epigenome tracks in 127 cell/tissue types including 8 epigenomic marks (H3K4me1, H3K4me3, H3K36me3, H3K27me3, H3K9me3, H3K27ac, H3K9ac and DNase I) from the ENCODE/Roadmap and then overlapped variants at each locus with a narrow peak of an annotation. Bayesian inference was performed to estimate the posterior probability of association (PPA) for each variant within the locus taking into account its epigenomic context.

### Functional mapping and annotation of top GWAS loci

We implemented FUMA v1.3.0 to functionally annotate and prioritize our GWAS results^34^. Specifically, information from 18 biological data repositories and tools was used to annotate variants with GWAS *p* < 5 × 10^−8^ and variants in LD with them (*r*^2^ ≥ 0.6) for each measure of cognitive function. Next, candidate genes were identified using positional mapping, eQTL mapping, and chromatin interaction mapping.

### Tissue-specific prediction of gene expression associated with cognitive function

We applied PrediXcan^35^ to predict and test for association of genetically regulated gene expression in brain tissues with each individual measure of cognitive function. We first estimated genetically regulated gene expression level in 10 brain tissues from each individual’s genotype using tissue-specific, whole-genome prediction model trained with reference transcriptome from the GTEx database. We then tested association between predicted tissue-specific gene expression with individual measures of cognitive function using the same LMM as was applied in the GWAS. Statistical significance was established using a Bonferroni correction.

### Gene-based and pathway analysis

Gene-based analysis using GWAS summary statistics was performed using fastBAT^36^ implemented in the GCTA. Pathway analysis was performed using DEPICT^37^ to prioritize genes, pathways and tissue/cell types.

### Shared genetic contribution to cognitive function and related cognitive traits

Using results from published GWAS, we generated polygenic risk scores (PRS) of cognitive function and related cognitive traits in the HCHS/SOL participants and estimated the shared genetic contribution among these traits using PRSice^38^. Summary statistics were obtained from GWAS of educational attainment^39^, general cognitive function^14^, reaction time^14^, major depression disorder^40^, neuroticism^41^, schizophrenia^42^, LOAD^43^, and MRI-defined white matter hyperintensities^44^ and hippocampal volume^45^. For each trait, the most predictive PRS was identified and regressed against each of the cognitive tests in our study, adjusting for fixed and random effects.

## Results

Table 1 shows the summary statistics of the sample in our analysis including sample size, distribution of phenotype, age, sex, and education level for each measure of cognitive function.

**Table 1:** Characteristics of participants for each individual measure of cognitive function.

	Total	Central American	Cuban	Dominican	Mexican	Puerto Rican	South American
*N* (% female)	7606 (61%)	762 (65%)	1506 (53%)	709 (65%)	2608 (63%)	1462 (60%)	559 (62%)
Mean age (SD)	55 (6)	55 (7)	56 (8)	55 (8)	55 (6)	56 (8)	55 (7)
% Education ≥ high school	58%	57%	72%	50%	48%	56%	75%
Mean score (SD)							
SIS (dichotomized)^a^	85%	88%	87%	77%	88%	78%	89%
B-SEVLT	8 (3)	9 (3)	8 (3)	8 (3)	9 (3)	7 (3)	9 (3)
WFT	18 (7)	18 (7)	18 (7)	16 (7)	19 (8)	17 (7)	21 (7)
DSST	34 (13)	30 (12)	34 (12)	27 (12)	35 (14)	36 (14)	36 (13)

^a^Proportion of participants with score of 5 or 6. *N* sample size, *SD* standard deviation, *SIS* six-item screener, B-SEVLT brief Spanish English verbal learning test, *WFT* word fluency test, *DSST* digit symbol substitution test.

**Fig. 1:**
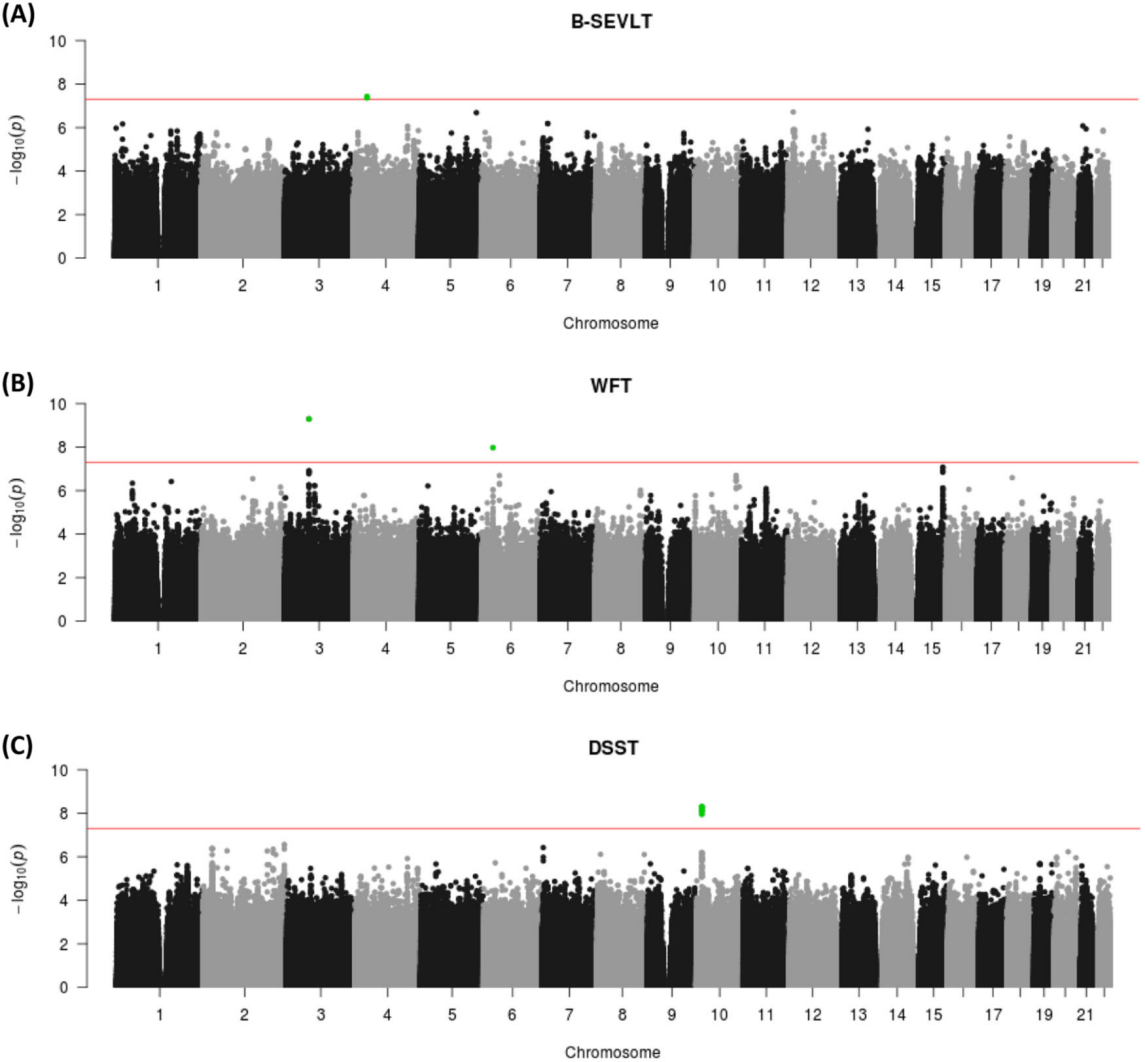
Manhattan plots of the genome-wide association study results for each cognitive test. **a** Brief Spanish English Verbal Learning Test (B-SEVLT); **b** Word Fluency Test (WFT); and **c** Digit Symbol Substitution Test (DSST). The red horizontal line represents the genome-wide significance threshold (*p* < 5 × 10^−8^) and the green dot represents the significant variants in each analysis.

We tested ~ 20 million SNVs and indels in up to 7600 US Hispanics for association with each measure of cognitive function. Potential population stratification of this diverse sample was well controlled (Supplementary Fig. 1). We identified one locus at 4p14 for B-SEVLT (lead SNV: rs113719683, *p* = 3.7 × 10^−8^) with three common SNVs (MAF = 6%) in high LD with each other (*r*^2^ ≥ 0.98); one common indel (rs59912956: MAF = 19%, *p* = 5.09 × 10^−10^) at 3p14.1 and one rare indel (rs568391433: MAF = 0.4%, *p* = 1.07 × 10^−8^) at 6p21.32 (*HLA* locus) for WFT; and one locus at 10p13 for DSST (lead SNV: rs74610382, *p* = 5.04 × 10^−9^) with six rare SNVs (MAF = 0.3%) in high LD with each other (*r*^2^ ≥ 0.78) (Fig. 1; Table 2). Adjustment for education level did not diminish the significance of the variants associated with B-SEVLT and WFT meaningfully, but had an impact on those associated with DSST, though they remained nominally significant (Table 2). Ancestry-specific allele frequency estimates showed that the three SNVs associated with B-SEVLT on chromosome 4 are observed most commonly in European ancestry; the rare indel associated with WFT is only observed in European ancestry. The common indel associated with WFT is observed in all 3 continental populations at similar frequency, while the six rare SNVs on chromosome 10 associated with DSST are observed only in African ancestry (Table 2). We tested these variants for replication in independent samples of ancestry corresponding to the most likely continental origin of the associated allele according to our estimated ancestry-specific allele frequencies, using the same or similar test that measure the same functional domain (Supplementary Table 2). The meta-analysis p-values in replication samples are shown in Table 2. Though none of these variants were replicated, the associations of the effect allele have the same direction comparing to those in the discovery sample (binomial test indicating such observation is not random *p* < 0.001).

**Table 2:** Genome-wide significant variants for each measure of cognitive function, their ancestry-specific allele frequency estimate, and replication in independent samples.

Test	Variant	chr	Position	Coded allele	Other allele	Coded allele frequency	*N*	Beta (se)	*P*-value	*P*-value (edu-adj)	Ancestry-specific allele frequency^a^			Replication *P*-value^b^
											Amerindian	European	African	
B-SEVLT	rs113719683	4	40433446	T	C	0.939	7562	0.6 (0.1)	3.70E-08	6.70E-08	0.997	**0.926**	1.000	0.800
	rs112178366	4	40433442	A	G	0.939	7562	0.6 (0.1)	4.06E-08	6.81E-08	0.997	**0.927**	1.000	0.709
	rs112927755	4	40433460	G	A	0.940	7562	0.6 (0.1)	4.32E-08	7.13E-08	0.997	**0.927**	1.000	0.775
WFT	rs59912956	3	69592250	GA	GAA	0.814	7446	1.0 (0.2)	5.09E-10	2.46E-09	0.815	0.828	0.805	0.434^c^
	rs568391433	6	33004530	C	CT	0.996	7446	−5.8 (1.0)	1.07E-08	5.09E-08	1.000	**0.995**	1.000	0.356^c^
DSST	rs74610382	10	16254360	G	A	0.997	7372	10.8 (1.8)	5.04E-09	1.32E-06	1.000	1.000	**0.985**	0.330
	rs116623781	10	16226698	G	A	0.997	7372	10.3 (1.8)	5.05E-09	2.05E-06	1.000	1.000	**0.984**	0.310
	rs181182078	10	16260622	A	T	0.997	7372	10.7 (1.8)	5.94E-09	1.88E-06	1.000	1.000	**0.985**	0.658
	rs150206471	10	16220142	C	T	0.997	7372	10.4 (1.8)	7.44E-09	6.27E-07	1.000	1.000	**0.984**	0.327
	rs142289140	10	16264277	C	G	0.997	7372	10.5 (1.8)	8.31E-09	2.91E-06	1.000	1.000	**0.985**	0.786
	rs115696258	10	16270189	C	T	0.997	7372	10.5 (1.8)	1.11E-08	3.47E-06	1.000	1.000	**0.986**	0.379

^a^Ethnic groups used in replication were determined by estimated ancestry-specific allele frequency (in bold).

^b^Replication *P*-values were generated by meta-analysis of ARIC, CARDIA and HRS. The direction of association with the effect allele for all variants are the same in the discovery and replication samples.

^c^The two variants for WFT were only observed in HRS so that the meta-analysis *P*-values were contributed solely by HRS. *B-SEVLT* brief Spanish English verbal learning test, *WFT* word fluency test, *DSST* digit symbol substitution test, *chr* chromosome, *N* sample size, *SE* standard error, *edu-adj* education adjusted, *ARIC* atherosclerosis risk in communities, *CARDIA* coronary artery risk development in young adults, *HRS* health and retirement study.

We also performed a look-up of our identified loci in the largest GWAS of general cognitive function (G) reported to date in a population of mostly European ancestry^14^. None of our genome-wide significant variants or their proxies were present in that GWAS, probably due to the filtering strategy used. However, because our identified SNPs may, themselves, not be causal but rather may tag (a) potential causal SNP(s) and because patterns of LD differ between Hispanics/Latinos and populations of European ancestry, direct look-up of our identified SNPs may not be informative or appropriate. Hence, we examined whether any SNPs located within 100 kb around our top SNPs showed evidence of association with G in Davies et al. (*p* < 0.05/4 loci = 1.25 × 10^−2^). For all 4 loci, we observed some evidence of association with general cognitive function (Supplementary Table 3). The strongest evidence was for the locus at 6p21.32 (108 SNPs; lowest *p* = 1.9 × 10^−4^, rs3129267).

Reciprocally, we looked up all 11 600 genome-wide significant variants reported in the largest GWAS of general cognitive function to date in the population of European ancestry representing 148 independent loci^29^, among which 8 variants from 2 loci reached the Bonferroni-corrected significance threshold (*p* < 3.4 × 10^−4^) (Supplementary Table 4).

To facilitate comparison of findings in this diverse population of Hispanics/Latinos with those reported in the largest GWAS of cognitive function to date^14^, we also performed a GWAS of general cognitive function (PC1) defined as in Davies et al^14^. No variant reached genome-wide significance although 14 variants in 6 loci reached the suggestive threshold of *p* < 5 × 10^−7^ (Supplementary Table 5). Seven of the 14 variants (4 loci) were directly observed in Davies et al. but did not show evidence of association with general cognitive function in our study. All but one locus harbored multiple SNPs within 100 kb of the top SNP, which showed evidence of association with G, with the strongest evidence for a locus at 12p12.3 (35 SNPs; lowest *p* = 3.5 × 10^−6^, rs7965359). Notably, the locus at 3p14.1 associated with WFT showed also evidence of association with G (rs59912956, *p* = 7.3 × 10^−8^).

Finally, we performed a meta-analysis of our general cognitive function GWAS with results from Davies et al^14^., which yielded 625 additional genome-wide significant variants (Supplementary Fig. 2). Among these, 160 were independent (*r*^2^ < 0.6) from the previously reported variants in Davies et al^14^. and represented 30 loci. Twenty-six of these loci (89 variants) showed low to moderate heterogeneity in the meta-analysis (*I*^2^ < 50%) (Supplementary Table 6).

We estimated the proportion of phenotypic variance explained by common variants for each measure of cognitive function as well as the pairwise phenotypic and genetic correlations among the cognitive measures (Table 3**)**. Individual measures of cognitive function show weak to moderate phenotypic correlation to each other (*r* = 0.19~0.47), whereas G was highly correlated with its component scores (*r* ≥ 0.7). Common variants explained a small proportion of the phenotypic variance for all measures of cognitive function (*h*^2^ = 0.08~ 0.31). Genetic correlations between B-SEVLT and WFT/DSST were not significant (*r* = 0.31 and 0.18, respectively). The remaining pairwise correlations were significant and showed a moderate to strong genetic correlation (*r* = 0.54~ 0.85).

**Table 3:**
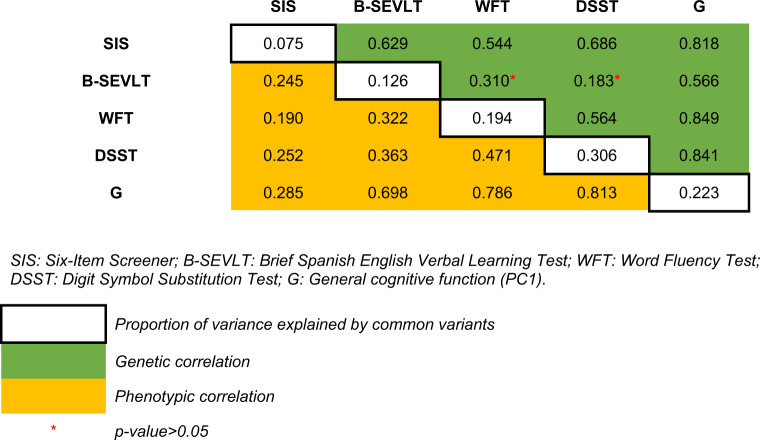
Heritability, genetic and phenotypic correlation estimate.

*SIS* six-item screener, *B-SEVLT* brief Spanish English verbal learning test, *WFT* word fluency test, *DSST* digit symbol substitution test, *G* general cognitive function (PC1).

**Fig. 2:**
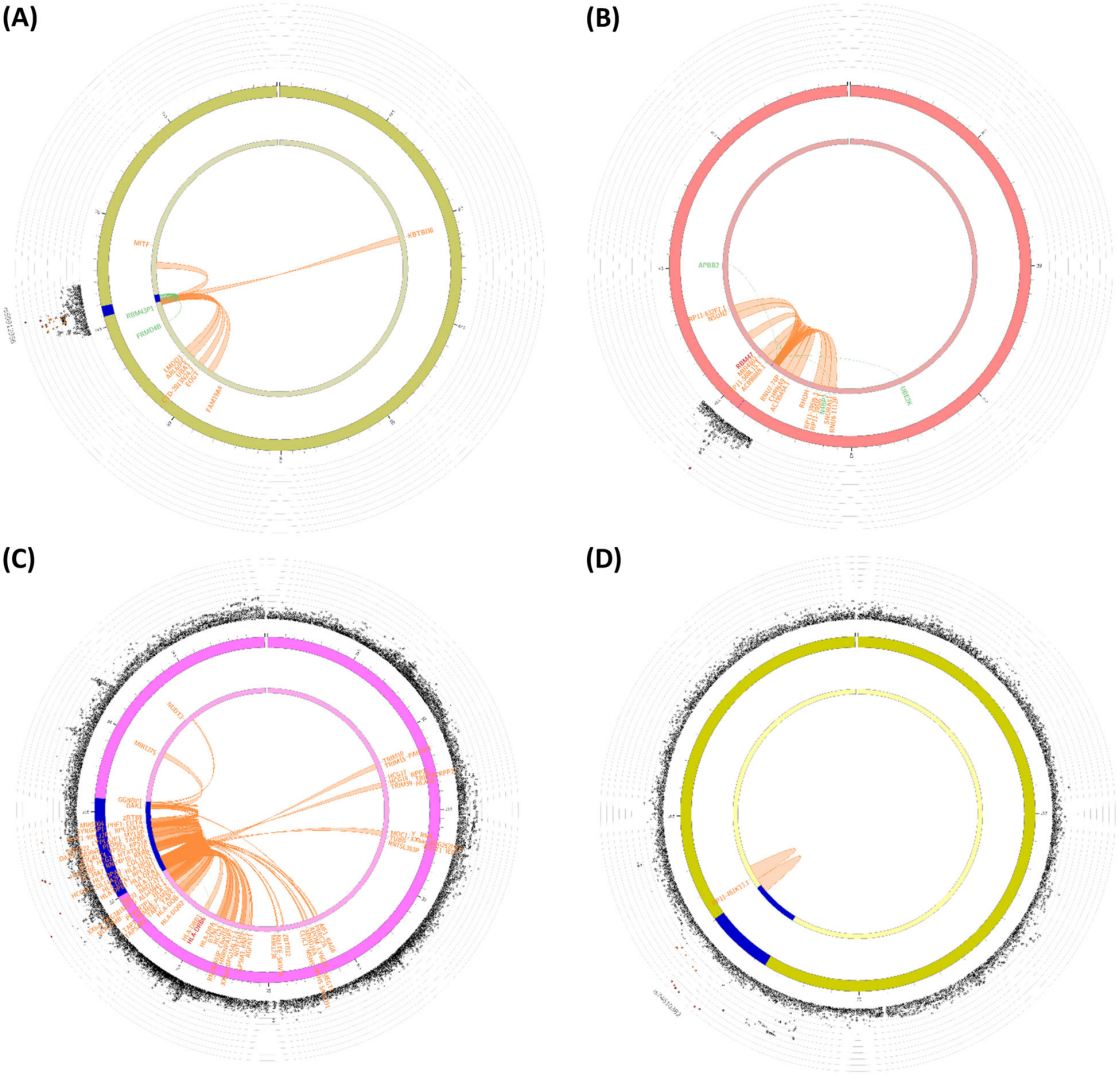
Circos plots showing genes linked to cognitive function at each associated locus. **a** locus at 4p14; **b** locus at 3p14; **c** locus at 6p21.32; and **d** locus at 10p13. Genes identified through expression quantitative trait locus (eQTL) mapping are shown in green and green lines connect an eQTL variant in the GWAS locus to its associated gene. Genes identified through chromatin-interaction mapping are shown in orange and orange lines connect regions of chromatin interaction. Genes identified through both eQTL and chromatin-interaction mapping are shown in red.

Functional annotation of the variants reaching genome-wide significance for cognitive measures in the present study is shown in Supplementary Table 7. All of the associated variants mapped to non-coding regions of the genome. To link the associated variants to genes, we applied the 3 gene-mapping strategies implemented in FUMA and summarized in Fig. 2. While these analyses are helpful to point to candidate genes with potential functional impact on cognitive function, higher credibility is given to those with convergent evidence from multiple analyses. For example, the SNVs at 4p14 associated with B-SEVLT mapped to intronic regions of the *RBM47* gene and were identified as eQTLs for 4 genes: *RBM47*, *APBB2*, *N4BP2* and *UBE2K*. In our PrediXcan analyses, the genetically-predicted gene expression of *UBE2K* in the basal ganglia was nominally associated with B-SEVLT (*p* = 0.036). Similarly, the variant at 6p21.32 associated with WFT mapped to the *HLA* region. Among the genes implicated by chromatin interaction mapping (Fig. 2), genetically-predicted gene expression of *HLA-DMB* in the frontal cortex was associated with WFT in our PrediXcan analyses (*p* = 0.019). The variant at 3p14.1 associated with WFT mapped upstream of *FRMD4B* and was in moderate LD (*r*^2^ = 0.6) with variants annotated as eQTLs of *FRMDB4* and the pseudogene *RBM43P1*. The SNVs at 10p13 associated with DSST mapped to an intergenic region. Chromatin interaction mapping implicated a gene encoding a lincRNA, *RP11-461K13.1*. Using RiVIERA, we inferred that among the six genome-wide significant rare variants at 10p13 for DSST, rs142289140 is more likely to be causal (PPA = 0.75), taking into account the epigenomic context at this locus (Supplementary Fig. 3).

In addition to selected genes implicated by our GWAS analyses, our PrediXcan analysis in multiple brain tissues showed no genes significantly associated with any of the cognitive measures after correcting for multiple comparisons, but we identified several suggestive associations. Notable findings include *IFT122* in the hippocampus for SIS (*p* = 8.13 × 10^−5^), *SNX31* in the basal ganglia for B-SEVLT (*p* = 3.74 × 10^−5^), *RPS6KB2* in the frontal cortex for WFT (*p* = 3.91 × 10^−5^), and *CSPG5* in the hypothalamus for DSST (*p* = 6.80 × 10^−5^) (Supplementary Fig. 4). No single variant within these genes, however, showed an association with the corresponding cognitive test (Supplementary Fig. 5).

The gene-based and pathway analyses did not yield significant results for any measure of cognitive function (Supplementary Table 8). Gene-set enrichment analysis using FUMA-implicated genes in all 4 GWAS loci showed a significant enrichment for genes previously reported in GWAS of autism spectrum disorder and schizophrenia (Supplementary Fig. 6).

PRS for educational attainment and general cognitive function were associated with all cognitive traits but explained little of the phenotypic variance in our sample. For example, the PRS for general cognitive function explained only a little than 1% of the variance of G (Supplementary Table 9). These results must be interpreted cautiously in light of the known complexity of PRS transferability and performance in non-European populations^46,47^.

## Discussion

We conducted a GWAS of multiple measures of cognitive function in a large and diverse sample of middle-aged and older US Hispanics/Latinos. We identified four novel loci encompassing genes previously implicated in neurological and psychiatric disorders. However, we were unable to convincingly establish an association of these loci with cognitive function in independent samples. In addition, few loci identified in populations of European ancestry demonstrated evidence of an association with cognitive function in this diverse sample of Hispanics/Latinos. Our study underscores the need for expanding genetic studies in this under-studied population and illustrates the challenges of replicating and interpreting findings in light of the paucity of genetic data from large and diverse Hispanic/Latino samples. Indeed, while GWAS have become the standard tool for exploring the genetic basis of human complex traits, as of 2019, Hispanic/Latino populations represent only 1% of individuals in these studies^48,49^. This is despite the recognition that increasing human subject diversity improves genetic discoveries^50^. Over 18% of the total US population are Hispanics/Latinos and their under-representation in genetic studies is likely to further exacerbate existing health disparities by limiting clinical applications of genetic research, such as risk prediction^48,49^. Acknowledging the complexity of PRS transferability and performance in non-European populations^46,47^, our finding that polygenic risk scores for general cognitive function or educational attainment estimated based on European ancestry data explain little of the variance in measures of cognitive function in diverse Hispanics/Latinos nonetheless further illustrates this argument.

Our study has considerable strengths. Considering the complexity of Hispanic/Latino ancestries, the multi-stage and multi-center sampling design of the HCHS/SOL made it the most diverse representative sample of the US Hispanic/Latino population to date. We used LMM and adjustment for “genetic-analysis group” to account for relatedness and genetic heterogeneity among ethnic groups. In this GWAS, we identified common and low frequency SNVs and indels associated with the performance in three cognitive tests that measure different functional domains in the brain. Inference of causal variants identified in this GWAS is challenging because GWAS signals are LD-based and mostly map within non-coding regions whose function is poorly understood. We integrated epigenomic and other functional annotations and derived predicted tissue-specific genetically regulated gene expression to identify relevant candidate genes.

Our GWAS of B-SEVLT, a measure of verbal learning and memory, identified three nearby and highly linked common SNVs in the intronic region of the gene *RBM47* at 4p14. This gene encodes RNA binding motif 47, a protein that plays an important role in the regulation of alternative splicing, mRNA stability, and RNA editing. Animal studies have shown that this RNA binding protein is critical for head formation during zebrafish embryogenesis^51^. Possible mechanisms that link *RBM47* to memory in primates are unknown. Intriguingly, a rare missense variant in *RBM47* has been associated with blood pressure and hypertension^52^, the most notable modifiable cardiovascular risk factor for both cognitive decline and dementia^53^. The identified variants at 4p14 were functionally linked to expression of another gene in the region, *UBE2K*, which encodes an ubiquitin-conjugating (E2) enzyme highly expressed in the brain. *UBE2K* has been implicated in the mediation of amyloid-β neurotoxicity and proteasome inhibition^54^. Its role in ubiquitinated protein accumulation and aggregation has been demonstrated in the pathology of several neurological diseases, including Huntington’s disease, Alzheimer’s disease, Parkinson’s disease, and schizophrenia^55–58^. The possibility that genetic variants at 4p14 may impact cognitive function through dysregulation of *UBE2K* function is in line with GWAS and other studies that implicate genes of the ubiquitin proteasome system in neurodegenerative diseases^59^. In addition, predicted genetically regulated expression of the gene *SNX31* in the basal ganglia is suggestively associated with B-SEVLT. *SNX31* encodes sorting nexin-31 which may be involved in protein trafficking. Whole exome sequencing detected a *SNX31* frameshift variant in a schizophrenia patient^60^. GWAS also identified suggestive association of *SNX31* variants with levels of clusterin and β-site APP cleaving enzyme in the cerebrospinal fluid^61,62^, which are, both, potential biomarkers of AD.

Two indels mapping to chromosome 3p14.1 were associated with WFT, a measure of executive and verbal functioning. One is common, located ~ 500 bp upstream of *FRMD4B*, and functionally linked to the expression of that gene. *FRMD4B* encodes FERM domain-containing protein 4B, a scaffolding protein that regulates epithelial cell polarity. This gene is one of the prominent hub genes within the myelination network implicated in LOAD^63^. Previous GWAS also identified suggestive associations between *FRMD4B* variants with LOAD^64^ and schizophrenia^65^. The other indel is a rare intergenic variant located in the *HLA* region at 6p21.32, which is a well-established risk locus underlying neurodegenerative diseases through neuroinflammation and immunoregulation^66^. Interestingly, each copy of the insertion (minor) predicts an increase in the test score of approximately 6 correct words produced, suggesting a possibly protective effect of this variant. In addition, predicted genetically regulated expression of the gene *RPS6KB2* in the frontal cortex is suggestively associated with WFT. *RPS6KB2* encodes ribosomal protein S6 kinase β-2, which phosphorylates specifically ribosomal protein S6, leading to an increase in protein synthesis and cell proliferation. *RPS6KB2* is a known gene of kinases involved in tau phosphorylation, and the common variation of this gene was associated with an increased risk and a later onset of AD^67^. Notably, frontal cortex has been recognized as the major brain structure that carries out fluency tasks^68^. Our findings suggest a potential effect of *RPS6KB2* on the executive and verbal functioning possibly through upregulation of a tau kinase in the frontal cortex.

A locus at 10p13 that encompasses six rare SNVs in high LD was identified for DSST, a measure of psychomotor speed and sustained attention. Each additional copy of the minor allele at each variant predicted a ~ 10 unit decrease in the test score. These SNVs are located in the intergenic region and are potentially functionally related to a lincRNA. Fine-mapping of this locus taking into account the epigenomic context further pointed to rs142289140 (*p* = 8.31 × 10^−9^, PPA = 0.75), a regulatory variant located at a CTCF-binding site. Interestingly, this site displays an epigenetic signature with the potential to be activated in H1-derived neuronal progenitor cultured cells^69^. Evidence is accumulating that CTCF-dependent gene expression regulation may play a role in brain aging. Animal studies suggested that neuronal CTCF is necessary for learning and memory^70^. Human studies also showed that genetic variants associated with neurodegenerative diseases are also enriched in CTCF-binding sites in brain tissues^71^. These data indicate that rare SNVs in the regulatory region at 10p13 may affect human cognition through regulation of CTCF-dependent gene expression. In addition, predicted genetically regulated expression of the gene *CSPG5* in the hypothalamus is suggestively associated with DSST. *CSPG5* encodes chondroitin sulfate proteoglycan 5, present exclusively in central nervous system tissues. This protein may function as a growth and differentiation factor involved in neuronal migration and neuritogenesis^72^. *CSPG5* has been previously implicated in schizophrenia^73^, and has been identified as a critical target of PHF6, the protein mutated in the intellectual disability disorder Börjeson–Forssman–Lehmann syndrome^74^. Reduced levels of brain-specific chondroitin sulfate proteoglycans, including *CSPG5*, were also associated with a delay in neurological development and the presence of a learning disability in early postnatal rats^75^.

Our GWAS failed to identify any genome-wide significant variants for SIS, a brief and reliable instrument that measures global mental status. The dichotomization of the original ordinal measure may have reduced power of our genetic studies. Predicted genetically regulated expression of the gene *IFT122* in the hippocampus is suggestively associated with SIS. *IFT122* encodes intra-flagellar transport protein 122 required for cilia formation during neuronal patterning. A genome-wide association analysis previously reported an association of an intronic variant of *IFT122* with the area of the left isthmus cingulate on neuroimaging, which potentially mediated an association of this variant with spatial orientation ability measured by the Pennsylvania line orientation test^76^. The association of hippocampal expression of this gene with SIS is in line with the previous implication of this gene in complex cognition.

Using the GCTA-GREML approach, we estimated the variance explained by common variants for each measure of cognitive function. Power calculation showed that we have at least 80% power to detect a heritability as low as 0.12. We estimated that common variants explain a small proportion of phenotypic variance for all cognitive tests, and this was further supported by our findings of multiple rare variants associated with different cognitive tests. We also showed that the genetic correlation between most of the pairs is moderate to strong. Of note, the observation of consistent moderate/strong genetic correlation of SIS with all other measures, albeit not the case for phenotypic correlation, suggests that SIS may still be a good global measure that covers a variety of cognitive functions in genetic studies. On the other hand, relatively low and non-significant correlation between B-SEVLT, WFT, and DSST indicates that they do not share much additive genetic variance attributable to common variants and thus partly explains why the significant findings are not shared among the multiple cognitive measures.

Several limitations of our study must also be acknowledged. Though our study was carefully designed and estimation of ancestry-specific allele frequency for the associated SNVs informed our choice of study population for replication, we were unable to replicate our genome-wide significant findings in independent samples. Possible reasons include an insufficient power of the replication samples due to limited sample size and differences in allele frequency; differences in the cognitive tests performed in the various replication samples, though to some extent they measure similar functional domains; the relatively young age of our discovery sample; differences in the genetic architecture at the associated loci due to complex local admixture, epistasis due to differences in genetic backgrounds, and gene-by-environment interaction that may vary among different populations. In addition, we cannot rule out that our findings may be false positives possibly due to residual or unmeasured confounding unique to this admixed and diverse population. Additional studies in other large samples of Hispanics/Latinos with comparable degree of diversity are therefore warranted for further validation of these findings and for a better understanding of the genetic architecture of this population.

To facilitate interpretation and comparison of our data with published results from a large sample of mostly European ancestry, we performed a GWAS of a measure of general cognitive function (PC1) as described by Davies et al^14^. No locus reached genome-wide significance likely due to low power. Six suggestive loci showed some limited evidence of association with general cognitive function in Davies et al. Notably one of the loci (3p14.1, *FRMD4B*) was the same as that identified in our GWAS of WFT. Only two out of 148 previously identified loci met the criteria for replication in our study. These include a locus on chromosome 4q24, which encompasses *TET2*, a gene encoding a DNA demethylase with known roles in the microglial inflammatory response and neurodegenerative diseases;^77,78^ and a locus on chromosome 13q31.2, which encompasses *LINC00433* but no protein-coding gene. Taken together, these results further illustrate the need for larger samples of diverse Hispanics/Latinos and the potentially unique genetic architecture of cognitive function in this population.

Although our sample size may have limited our independent discoveries, meta-analysis of our data with the published GWAS of general cognitive function in a European ancestry sample yielded 30 additional loci reaching genome-wide significance. Among these, 26 showed little evidence of heterogeneity among the 2 studies, suggesting the possibility of shared genetic susceptibility among Hispanics/Latinos and those of European ancestry.

In conclusion, we report the results of a large-scale GWAS of cognitive function among diverse middle-aged and older US Hispanics/Latinos. We identified genome-wide significant common and rare variants associated with multiple measures of cognitive function, indicating possible candidate genes. Replication in independent Hispanic/Latino samples with the comparable level of diversity are warranted to confirm our findings. Our study underscores the pressing need for genetic investigations in large samples of Hispanics/Latinos in order to characterize the genetic underpinnings of cognitive function, which are both unique to this population and shared with populations of other ancestries.

## Supplementary information

Supplementary Information

## Data Availability

Genotype and imputed data of the HCHS/SOL can be requested via dbGaP study accession phs000880. Phenotype data can be requested via dbGaP study accession phs000810. Summary statistics for the GWAS are available upon request to the authors.
