# Supplementary material for: Genome-wide association study of cognitive function in diverse Hispanics/Latinos: results from the Hispanic Community Health Study/Study of Latinos
Source: Transl Psychiatry. 2020 Jul 22;10:245. doi: 10.1038/s41398-020-00930-2 (PMC7376098; doi:10.1038/s41398-020-00930-2)
Supplement: Supplementary file 1 — Supplementary Information [file 41398_2020_930_MOESM1_ESM.docx]

**SUPPLEMENTARY INFORMATION**

Genome-wide association study of cognitive function in diverse Hispanics/Latinos: the Hispanic Community Health Study / Study of Latinos

- Supplementary Methods……………………………………………………………………….……………………………………2
- Supplementary Acknowledgements……………………………………………………………………………………………4
- Supplementary References…………………………………………………………………….……………………………………5
- Supplementary Tables…………………………………………………………………………………………………………………6
- Supplementary Figures………………………………………………………………………….…………………….……………16

**Supplementary Methods**

Atherosclerosis Risk in Communities (ARIC) study

The ARIC study is a population-based cohort study of atherosclerosis and clinical atherosclerotic diseases.^1^ At its inception (1987-1989), 15,792 individuals aged 45-64 years were recruited from four U.S. communities: suburban Minneapolis, MN; Washington County, MD; Forsyth County, NC; and Jackson, MS. Since the baseline exam, five subsequent follow-up visits have been carried out, among which neurocognitive functioning was assessed in visit 2 (1990-1992), visit 4 (1996-1998), visit 5 (2011-2013), and visit 6 (2016 – 2017) including the Delayed Word Recall Test (DWRT), the Digit Symbol Substitution Test (DSST), and the Word Fluency Test (WFT). In this study the test scores from visit 2 were used. A summary of each test as well as the demographic information of the ARIC replication sample can be found in **Supplementary Table 2**.

At baseline, blood was drawn for DNA extraction and participants consented to genetic testing. Genome-wide genotyping was conducted at the Broad Institute using the Affymetrix 6.0 SNP Array. Genotyping calling was performed using Birdseed for 9,747 European Americans and 3,207African Americans. Imputation was performed on the QC’ed data in two steps: pre-phasing with SHAPEIT (v1.r532) and imputation with IMPUTE2, using the 1000 Genomes Phase I reference panel. After QC, there were 9,347,320 SNPs in European Americans and 15,898,763 SNPs in African Americans.

Coronary Artery Risk Development in Young Adults (CARDIA) study

The CARDIA study is a population based, prospective cohort examining the development and determinants of clinical and subclinical cardiovascular disease and its risk factors.^2^ The CARDIA study initial enrollment (1985-1986) consisted of 5,115 European Americans and African American men and women between 18 and 30 years old. The study is multicenter with recruitment in Birmingham, AL; Chicago, IL; Minneapolis, MN; and Oakland, CA. Baseline measurements were repeated, and additional measurements performed, at Years 2, 5, 7, 10, 15, 20, 25, and 30. At the Year 25 (2010-2011) exam, multiple cognitive tests were carried out to measure cognitive function, among which the Rey Auditory Verbal Learning Test (RAVLT) was used in this study. A summary of RAVLT as well as the demographic information of the CARDIA replication sample can be found in **Supplementary Table 2**.

Genotyping for the CARDIA white participants was performed at the Broad Institute using the Affymetrix 6.0 SNP Array. Genotype calls were produced using BEAGLECALL (EA) or Birdseeed (AA). The 1000 Genomes imputation was implemented using BEAGLE v3.3.2 (EA) or Minimac v2013.7.17 (AA) based on the cosmopolitan panel from Phase I Integrated Release Version 3 Haplotypes (2010-11 data freeze, 2012-03-14 haplotypes). After QC, there were 7,544,843 SNPs in EA and 15,562,229 SNPs in AA.

Health and Retirement Study (HRS)

The HRS is a longitudinal survey of a representative sample of Americans over the age of 50 initiated in 1992.^3^ The current sample is over 26,000 persons in 17,000 households. Respondents are interviewed every two years about income and wealth, health and use of health services, work and retirement, and family connections. Three cognition outcomes were determined to be reasonably equivalent to those used in the HCHS/SOL, including the Telephone Interview for Cognitive Status (TICS), the Immediate and Delayed Recall Test (RECALL), and the Animal Fluency Test (AFT). A summary of each test as well as the demographic information of the HRS replication sample can be found in **Supplementary Table 2**.

DNA was extracted from saliva collected during a face-to-face interview in the respondents' homes. These data represent respondents who provided DNA samples and signed consent forms in 2006, 2008, 2010, and 2012. SNP genotyping was performed using the Illumina Omni2.5 Beadchip. Pre-phasing for imputation was performed using SHAPEIT2. Imputation to 1000 Genomes Phase III integrated variant set (v5, initial release in May 2013, haplotype release in Oct 2014) was performed using the Minimac3 on the Michigan Imputation Server.

**URLs**

R, <https://www.r-project.org/>;

GENESIS, <https://bioconductor.org/packages/release/bioc/html/GENESIS.html>;

ASAFE, <https://bioconductor.org/packages/release/bioc/html/ASAFE.html>;

GCTA, <http://cnsgenomics.com/software/gcta/>;

RiVIERA, <https://github.com/yueli-compbio/RiVIERA>;

ENCODE/Roadmap narrow peak calls, <https://egg2.wustl.edu/roadmap/web_portal/processed_data.html>;

PrediXcan, <https://github.com/hakyimlab/PrediXcan>;

GTEx prediction models, <http://predictdb.org/>;

DEPICT, <https://github.com/perslab/depict>;

PRSice, <https://github.com/choishingwan/PRSice>;

FUMA GWAS, <https://fuma.ctglab.nl/>

GWAS of general cognitive function / reaction time, <http://www.ccace.ed.ac.uk/node/335>; educational attainment / cognitive performance / neuroticism / schizophrenia, <https://www.thessgac.org/data>; major depression disorder, <https://www.med.unc.edu/pgc/results-and-downloads>; LOAD, <http://web.pasteur-lille.fr/en/recherche/u744/igap/igap_download.php>; white matter hyperintensities / hippocampal volume, <https://drive.google.com/drive/folders/0BzYDtCo_doHJNHdPMUdOYzliSjA?ogsrc=32>

**Supplementary Acknowledgements**

The **Atherosclerosis Risk in Communities** (ARIC) study has been funded in whole or in part with Federal funds from the National Heart, Lung, and Blood Institute, National Institutes of Health, Department of Health and Human Services (contract numbers HHSN268201700001I, HHSN268201700002I, HHSN268201700003I, HHSN268201700004I and HHSN268201700005I), R01HL087641, R01HL059367 and R01HL086694; National Human Genome Research Institute contract U01HG004402; and National Institutes of Health contract HHSN268200625226C. The authors thank the staff and participants of the ARIC study for their important contributions. Infrastructure was partly supported by Grant Number UL1RR025005, a component of the National Institutes of Health and NIH Roadmap for Medical Research.

The **Coronary Artery Risk Development in Young Adults** (CARDIA) is conducted and supported by the National Heart, Lung, and Blood Institute (NHLBI) in collaboration with the University of Alabama at Birmingham (HHSN268201800005I & HHSN268201800007I), Northwestern University (HHSN268201800003I), University of Minnesota (HHSN268201800006I), and Kaiser Foundation Research Institute (HHSN268201800004I). Genotyping was funded as part of the NHLBI Candidate-gene Association Resource (N01-HC-65226) and the NHGRI Gene Environment Association Studies (GENEVA) (U01-HG004729, U01-HG04424, and U01-HG004446). This project was also supported by grant R01 HL122658. This manuscript has been reviewed by CARDIA for scientific content.

The **Health and Retirement Study** (HRS) is supported by NIA grant U01AG009740. Analysis of cognitive phenotypes is also supported by NIA grant R03AG048806. Genotyping was funded separately by NIA grants RC2AG036495 and RC4AG039029. Genotyping was conducted by the NIH Center for Inherited Disease Research (CIDR) at Johns Hopkins University. Genotyping quality control and final preparation of the data were performed by the University of Michigan.

**Supplementary References**

1 The ARIC Investigators. The Atherosclerosis Risk in Communities (ARIC) Study: design and objectives. *Am J Epidemiol* 1989; **129**: 687–702.

2 Friedman GD, Cutter GR, Donahue RP, Hughes GH, Hulley SB, Jacobs DR *et al.* CARDIA: study design, recruitment, and some characteristics of the examined subjects. *J Clin Epidemiol* 1988; **41**: 1105–1116.

3 Sonnega A, Faul JD, Ofstedal MB, Langa KM, Phillips JWR, Weir DR. Cohort Profile: the Health and Retirement Study (HRS). *Int J Epidemiol* 2014; **43**: 576–585.

**Supplementary Tables**

**Supplementary Table 1. Description of cognitive tests in the study.**

| Test | Functional domain measured | Test procedure | Scoring | Score range |
| --- | --- | --- | --- | --- |
| SIS | Global mental status | Repeat three words and answer three orientation questions | Count the number of correct responses | 0 - 6 |
| B-SEVLT | Verbal learning and memory | After recalling a list of 15 common words over three trials, recall the same list of 15 words after a short delay during which a distracter list of words is presented | Count the number of correctly recalled words | 0 - 15 |
| WFT | Executive and verbal functioning | Produce as many words as possible that begin with the letters F and A within 60 seconds respectively | Count the number of correctly produced words beginning with both letters | 0 - |
| DSST | Psychomotor speed and sustained attention | Substitute numbers with symbols as many as possible using a key provided by the examiner within 90 seconds | Count the number of correct digit-symbol substitutions | 0 - 93 |

*SIS: Six-Item Screener; B-SEVLT: Brief Spanish English Verbal Learning Test; WFT: Word Fluency Test; DSST: Digit Symbol Substitution Test.*

**Supplementary Table 2. Description of the cognitive tests and characteristics of the replication samples.**

| Study | Test | Test procedure | Scoring | Score range | Test approximated | European | | African | | Hispanics | |
| --- | --- | --- | --- | --- | --- | --- | --- | --- | --- | --- | --- |
|  |  |  |  |  |  | **N** | **Score mean±SD** | **N** | **Score mean±SD** | **N** | **Score mean±SD** |
| ARIC | DWRT | Recall a list of ten common nouns five minutes after the learning | Count the number of correctly recalled nouns | 0 - 10 | B-SEVLT | 8995 | 7±1 | 1676 | 6±2 | NA | |
|  | WFT | Produce as many words as possible that begin with the letters F, A, and S within 60 seconds respectively | Count the number of correctly produced words beginning with all letters | 0 - | WFT | 7410 | 35±12 | 1673 | 30±13 |  |  |
|  | DSST | Substitute numbers with symbols as many as possible using a key provided by the examiner within 90 seconds | Count the number of correct digit-symbol substitutions | 0 - 93 | DSST | 7407 | 50±11 | 1670 | 33±13 |  |  |
| CARDIA | RAVLT | After recalling a list of 15 common words over five trials, recall the same list of 15 words after 10-minute delay during which a distracter list of words is presented | Count the number of correctly recalled words | 0 - 15 | B-SEVLT | 1398 | 9±3 | 705 | 7±3 | NA | |
| HRS | TICS | Date naming, object naming, president/vice president naming, backwards count, serial 7's | Count the number of correct responses | 0 - 15 | SIS | 5485 | 13±2 | 780 | 11±2 | 580 | 12±2 |
|  | RECALL | Immediate and delayed recall of 10 nouns | Count the number of correctly recalled words | 0 - 20 | B-SEVLT | 10 503 | 11±3 | 2531 | 9±3 | 2064 | 9±3 |
|  | AFT | Produce as many animal names as possible within 60 seconds respectively | Count the number of correctly produced animal names | 0 - | WFT | 2677 | 20±7 | 1364 | 16±6 | 1129 | 17±7 |

*ARIC: Atherosclerosis Risk in Communities; CARDIA: Coronary Artery Risk Development in Young Adults; HRS: Health and Retirement Study; DWRT: Delayed Word Recall Test; WFT: Word Fluency Test; DSST: Digit Symbol Substitution Test; RAVLT: Rey Auditory Verbal Learning Test; TICS: Telephone Interview for Cognitive Status; RECALL: Immediate and Delayed Recall Test; AFT: Animal Fluency Test; B-SEVLT: Brief Spanish English Verbal Learning Test; SIS: Six-Item Screener; N: sample size; SD: standard deviation.*

**Supplementary Table 3. Look-up of our genome-wide significant loci for individual cognitive tests in the Davies et al (2018) GWAS of general cognitive function.**

| Test | Locus | N variants* | Variant with lowest p-value | P-value |
| --- | --- | --- | --- | --- |
| B-SEVLT | 4p14 | 31 | rs4504294 | 3.49E-04 |
| WFT | 3p14.1 | 47 | rs183163944 | 2.15E-04 |
|  | 6p21.32 | 108 | rs3129267 | 1.86E-04 |
| DSST | 10p13 | 68 | rs2355635 | 1.11E-03 |

****Number of variants located within ±100 kb of our top identified variants at each locus for the corresponding cognitive test that show evidence of association with general cognitive function in the Davies et al (2018) GWAS (p<0.0125). B-SEVLT: Brief Spanish English Verbal Learning Test; WFT: Word Fluency Test; DSST: Digit Symbol Substitution Test.*

**Supplementary Table 4. Genome-wide significant variants for general cognitive function (Davies et al 2018) that reached significant threshold (p < 3.4×10^−4^) in the present study.**

| Variant | chr | Position | Coded allele | Other allele | Davies et al 2018 | | |  | HCHS/SOL | | | |
| --- | --- | --- | --- | --- | --- | --- | --- | --- | --- | --- | --- | --- |
|  |  |  |  |  | **Coded allele frequency** | **Z** | **P-value** |  | **Coded allele frequency** | **Beta** | **P-value** | **Trait** |
| rs2726513 | 4 | 106217358 | T | G | 0.407 | -8.6 | 5.26E-18 |  | 0.380 | -0.5 | 1.33E-04 | WFT |
| rs2726482 | 4 | 106261006 | A | G | 0.418 | 7.7 | 1.75E-14 |  | 0.442 | 0.4 | 3.01E-04 | WFT |
| rs10010325 | 4 | 106106353 | A | C | 0.486 | 5.9 | 4.33E-09 |  | 0.487 | 0.2 | 2.62E-04 | B-SEVLT |
| rs9520384 | 13 | 89016992 | A | G | 0.313 | -5.8 | 5.62E-09 |  | 0.462 | -0.5 | 2.85E-05 | WFT |
|  |  |  |  |  |  |  |  |  |  | -0.08 | 1.28E-04 | G |
| rs1117483 | 13 | 89020093 | A | T | 0.314 | -5.8 | 6.06E-09 |  | 0.462 | -0.5 | 3.08E-05 | WFT |
|  |  |  |  |  |  |  |  |  |  | -0.08 | 1.44E-04 | G |
| rs7327009 | 13 | 89038581 | C | G | 0.700 | 5.6 | 1.61E-08 |  | 0.550 | 0.5 | 3.61E-05 | WFT |
|  |  |  |  |  |  |  |  |  |  | 0.08 | 1.61E-04 | G |
| rs978165 | 13 | 89033066 | T | C | 0.699 | 5.6 | 1.61E-08 |  | 0.548 | 0.5 | 4.15E-05 | WFT |
|  |  |  |  |  |  |  |  |  |  | 0.08 | 2.34E-04 | G |
| rs7318918 | 13 | 89037467 | A | G | 0.299 | -5.6 | 2.01E-08 |  | 0.449 | -0.5 | 3.17E-05 | WFT |
|  |  |  |  |  |  |  |  |  |  | -0.08 | 1.34E-04 | G |

*chr: chromosome; HCHS/SOL: Hispanic Community Health Study / Study of Latinos; WFT: Word Fluency Test; B-SEVLT: Brief Spanish English Verbal Learning Test; G: general cognitive function.*

**Supplementary Table 5. Results and look-up of our general cognitive function GWAS suggestive variants (p < 5×10^−7^) in Davies et al (2018)**

| Locus | Variant | chr | Position | Coded  allele | Other  allele | Coded  allele  frequency | Beta | SE | p.SOL | p.Davies | N  Variants* | Variant with  lowest p-value | P-value |
| --- | --- | --- | --- | --- | --- | --- | --- | --- | --- | --- | --- | --- | --- |
| 2q24.2 | rs113219181 | 2 | 160024713 | A | G | 0.99 | -0.58 | 0.12 | 4.02E-07 | 0.24 | 18 | rs138148716 | 1.82E-03 |
| 3p14.1 | rs59912956 | 3 | 69592250 | GA | GAA | 0.81 | 0.14 | 0.03 | 7.35E-08 | NA | 32 | rs183163944 | 2.15E-04 |
|  | rs12633166 | 3 | 69599093 | A | C | 0.82 | 0.13 | 0.03 | 4.96E-07 | NA |  |  |  |
| 9p24.1 | rs10815808 | 9 | 8262329 | C | T | 0.43 | -0.11 | 0.02 | 5.57E-08 | 0.60 | 158 | rs56039946 | 6.06E-04 |
| 12p12.3 | rs1918307 | 12 | 16014695 | T | C | 0.36 | 0.11 | 0.02 | 2.42E-07 | 0.17 | 35 | rs7965359 | 3.51E-06 |
|  | rs10846241 | 12 | 16024173 | A | G | 0.36 | 0.11 | 0.02 | 3.44E-07 | 0.15 |  |  |  |
|  | rs7135906 | 12 | 16033778 | G | C | 0.36 | 0.11 | 0.02 | 3.91E-07 | 0.15 |  |  |  |
| 14q31.3 | rs78071306 | 14 | 89561868 | T | G | 0.98 | 0.40 | 0.08 | 4.78E-07 | NA | 17 | rs7153598 | 1.22E-04 |
|  | rs77359253 | 14 | 89562184 | G | A | 0.98 | 0.40 | 0.08 | 4.72E-07 | NA |  |  |  |
|  | rs7152923 | 14 | 89565825 | G | A | 0.98 | 0.40 | 0.08 | 4.58E-07 | NA |  |  |  |
|  | rs145264452 | 14 | 89568189 | C | T | 0.98 | 0.40 | 0.08 | 4.14E-07 | NA |  |  |  |
|  | rs144557525 | 14 | 89573253 | C | T | 0.99 | 0.47 | 0.09 | 1.11E-07 | NA |  |  |  |
| 18q21.2 | rs139987615 | 18 | 48581979 | T | C | 0.98 | -0.35 | 0.07 | 8.02E-08 | 0.60 | 0 | NA | NA |
|  | rs77532548 | 18 | 48629383 | G | A | 0.97 | -0.33 | 0.06 | 1.26E-07 | 0.58 |  |  |  |

**Number of variants located within ±100 kb of our top variants at each locus that show evidence of association with general cognitive function in the Davies et al (2018) GWAS (p<0.0083). chr: chromosome;* *SE: standard error; p.SOL: p-value in the present study; p.Davies: p-value in the Davies et al (2018) study.*

**Supplementary Table 6. Additional variants reaching genome-wide significance threshold for general cognitive function by meta-analyzing the present study with non-significant variants in the Davies et al (2018) GWAS that are independent of their significant nearby variants and shows low to moderate heterogeneity in the meta-analysis.**

| Variant | chr | Position | Coded allele | Other allele | p.Meta | Direction | p.Davies | p.SOL |
| --- | --- | --- | --- | --- | --- | --- | --- | --- |
| rs17008991 | 2 | 73610116 | A | G | 4.59E-08 | -- | 1.58E-07 | 6.35E-02 |
| rs111845822 | 2 | 73627310 | A | G | 4.78E-08 | -- | 1.54E-07 | 7.50E-02 |
| rs62176229 | 2 | 156216988 | C | G | 4.44E-08 | ++ | 7.65E-08 | 2.94E-01 |
| rs10497818 | 2 | 199498173 | A | T | 4.45E-08 | ++ | 9.08E-08 | 2.13E-01 |
| rs9973442 | 2 | 199522858 | A | C | 4.41E-08 | -- | 7.88E-08 | 2.76E-01 |
| rs11131096 | 3 | 7923576 | A | C | 1.77E-08 | ++ | 8.72E-08 | 2.42E-02 |
| rs4860797 | 4 | 67801134 | A | G | 3.63E-08 | ++ | 1.39E-07 | 4.73E-02 |
| rs6830599 | 4 | 67875009 | A | C | 4.02E-08 | ++ | 8.58E-08 | 1.95E-01 |
| rs6831786 | 4 | 67875548 | A | C | 4.13E-08 | ++ | 8.83E-08 | 1.94E-01 |
| rs13108103 | 4 | 67876929 | T | C | 3.92E-08 | -- | 8.65E-08 | 1.81E-01 |
| rs183720171 | 5 | 64915685 | A | G | 2.90E-08 | -- | 1.79E-07 | 1.99E-02 |
| rs4710962 | 6 | 21031435 | T | C | 1.43E-08 | -- | 5.61E-08 | 4.81E-02 |
| rs6569077 | 6 | 98212409 | T | C | 2.84E-08 | ++ | 5.58E-08 | 2.32E-01 |
| rs13197257 | 6 | 128333682 | T | G | 2.96E-08 | ++ | 6.13E-08 | 2.09E-01 |
| rs2883598 | 7 | 11907220 | A | G | 3.78E-08 | ++ | 1.10E-07 | 9.70E-02 |
| rs76219962 | 7 | 105084781 | A | G | 3.66E-08 | -- | 1.25E-07 | 7.37E-02 |
| rs2920470 | 8 | 92977484 | T | C | 3.69E-08 | ++ | 1.23E-07 | 6.96E-02 |
| rs2979856 | 8 | 92980145 | A | T | 4.83E-08 | -- | 1.48E-07 | 8.50E-02 |
| rs2976502 | 8 | 92980734 | A | G | 4.84E-08 | -- | 1.73E-07 | 5.67E-02 |
| rs2920462 | 8 | 92985508 | A | G | 4.29E-08 | ++ | 1.39E-07 | 7.44E-02 |
| rs2920464 | 8 | 92987753 | T | C | 2.88E-08 | ++ | 1.29E-07 | 3.07E-02 |
| rs1838181 | 8 | 92993679 | A | G | 4.15E-08 | -- | 1.35E-07 | 7.37E-02 |
| rs2920465 | 8 | 92996143 | T | C | 4.07E-08 | -- | 1.62E-07 | 4.26E-02 |
| rs1443559 | 8 | 92997242 | A | G | 3.84E-08 | -- | 1.40E-07 | 5.45E-02 |
| rs1899666 | 8 | 93006087 | A | G | 4.43E-08 | -- | 1.65E-07 | 5.09E-02 |
| rs7825562 | 8 | 93022696 | T | C | 3.48E-08 | -- | 1.61E-07 | 2.73E-02 |
| rs6986808 | 8 | 93024393 | A | C | 4.08E-08 | ++ | 1.61E-07 | 4.31E-02 |
| rs7844067 | 8 | 93026078 | T | C | 3.52E-08 | -- | 1.48E-07 | 3.63E-02 |
| rs7827887 | 8 | 93026374 | A | G | 2.84E-08 | ++ | 1.37E-07 | 2.48E-02 |
| rs28716374 | 8 | 93030411 | T | G | 3.93E-08 | -- | 1.63E-07 | 3.74E-02 |
| rs11779617 | 8 | 93034215 | A | G | 4.60E-08 | -- | 2.03E-07 | 3.08E-02 |
| rs13250170 | 8 | 93035331 | A | G | 4.64E-08 | ++ | 1.71E-07 | 5.18E-02 |
| rs7820440 | 8 | 93036627 | T | C | 3.72E-08 | ++ | 1.76E-07 | 2.53E-02 |
| rs9297901 | 8 | 93036795 | T | C | 4.36E-08 | ++ | 1.80E-07 | 3.78E-02 |
| rs4623479 | 8 | 93038708 | T | C | 2.88E-08 | ++ | 1.33E-07 | 2.81E-02 |
| rs4623480 | 8 | 93038787 | T | C | 3.04E-08 | ++ | 1.40E-07 | 2.81E-02 |
| rs6988317 | 8 | 93042221 | A | G | 4.98E-08 | ++ | 1.51E-07 | 8.62E-02 |
| rs11785362 | 8 | 93045784 | A | G | 4.78E-08 | -- | 2.01E-07 | 3.54E-02 |
| rs72671424 | 8 | 93056264 | A | G | 4.87E-08 | ++ | 2.06E-07 | 3.47E-02 |
| rs35983766 | 8 | 93056882 | T | C | 4.97E-08 | ++ | 1.86E-07 | 4.93E-02 |
| rs4735044 | 8 | 93057478 | T | C | 4.64E-08 | ++ | 1.74E-07 | 4.93E-02 |
| rs13259499 | 8 | 93057823 | A | G | 4.92E-08 | ++ | 2.03E-07 | 3.73E-02 |
| rs36005889 | 8 | 93058638 | A | C | 4.52E-08 | ++ | 1.66E-07 | 5.24E-02 |
| rs4477079 | 8 | 93059038 | T | C | 4.84E-08 | -- | 1.75E-07 | 5.43E-02 |
| rs734113 | 8 | 93060095 | T | C | 4.66E-08 | ++ | 1.71E-07 | 5.25E-02 |
| rs746142 | 8 | 93060566 | T | C | 4.87E-08 | -- | 1.96E-07 | 4.00E-02 |
| rs10092162 | 8 | 93062830 | A | C | 4.61E-08 | ++ | 1.86E-07 | 4.01E-02 |
| rs7007307 | 8 | 93063802 | A | C | 3.72E-08 | ++ | 1.41E-07 | 4.87E-02 |
| rs7833370 | 8 | 93066653 | C | G | 4.38E-08 | ++ | 1.68E-07 | 4.66E-02 |
| rs1410543 | 9 | 83217361 | C | G | 4.10E-08 | ++ | 6.66E-08 | 3.28E-01 |
| rs1410542 | 9 | 83217377 | A | G | 4.20E-08 | ++ | 6.62E-08 | 3.45E-01 |
| rs10902976 | 10 | 125446938 | A | C | 2.49E-08 | ++ | 9.40E-08 | 5.07E-02 |
| rs2203596 | 10 | 125449415 | A | G | 1.84E-08 | ++ | 7.97E-08 | 3.55E-02 |
| rs7084727 | 10 | 125451208 | A | G | 1.48E-08 | -- | 5.67E-08 | 5.12E-02 |
| rs7907303 | 10 | 125458081 | A | T | 4.07E-08 | ++ | 1.07E-07 | 1.23E-01 |
| rs2114674 | 10 | 125458992 | T | C | 4.08E-08 | ++ | 1.14E-07 | 1.07E-01 |
| rs2162533 | 10 | 125459217 | A | C | 3.50E-08 | ++ | 1.11E-07 | 7.87E-02 |
| rs11248236 | 10 | 125459587 | T | C | 4.64E-08 | -- | 1.29E-07 | 1.07E-01 |
| rs10789907 | 11 | 112559814 | A | C | 3.69E-08 | -- | 9.28E-08 | 1.37E-01 |
| rs2066591 | 13 | 50708904 | T | C | 4.76E-08 | -- | 1.72E-07 | 5.45E-02 |
| rs1932232 | 13 | 88974732 | A | G | 3.75E-08 | -- | 1.11E-07 | 9.34E-02 |
| rs9555294 | 13 | 88975146 | A | G | 4.30E-08 | -- | 1.31E-07 | 8.62E-02 |
| rs9558895 | 13 | 88975313 | T | C | 4.25E-08 | -- | 1.27E-07 | 9.02E-02 |
| rs1041690 | 13 | 88977011 | C | G | 4.93E-08 | -- | 1.68E-07 | 6.38E-02 |
| rs9520333 | 13 | 89007864 | T | C | 3.57E-08 | -- | 1.33E-07 | 5.13E-02 |
| rs7143269 | 14 | 37022816 | A | G | 1.75E-08 | ++ | 8.21E-08 | 2.83E-02 |
| rs1956961 | 14 | 37031500 | A | T | 1.06E-08 | ++ | 5.14E-08 | 2.67E-02 |
| rs427067 | 14 | 37041396 | T | C | 1.20E-08 | -- | 5.43E-08 | 3.24E-02 |
| rs963120 | 14 | 37070214 | T | C | 4.59E-08 | -- | 1.63E-07 | 5.73E-02 |
| rs111507750 | 14 | 37081012 | A | G | 4.57E-08 | -- | 1.38E-07 | 8.77E-02 |
| rs1035738 | 15 | 52196830 | T | C | 2.72E-08 | ++ | 5.12E-08 | 2.54E-01 |
| rs72739469 | 15 | 65738080 | T | C | 3.17E-08 | -- | 1.14E-07 | 6.44E-02 |
| rs11645302 | 16 | 28951457 | T | C | 2.99E-08 | ++ | 1.41E-07 | 2.88E-02 |
| rs4788115 | 16 | 28998111 | A | T | 2.47E-08 | ++ | 1.02E-07 | 4.16E-02 |
| rs67361341 | 19 | 31842002 | A | C | 3.06E-08 | -- | 1.17E-07 | 4.83E-02 |
| rs8109639 | 19 | 31928464 | A | G | 2.65E-08 | -- | 8.06E-08 | 8.87E-02 |
| rs1593085 | 19 | 31929234 | A | G | 4.40E-08 | -- | 1.21E-07 | 1.10E-01 |
| rs8103449 | 19 | 31930388 | A | T | 4.84E-08 | -- | 1.26E-07 | 1.25E-01 |
| rs7256476 | 19 | 31931005 | T | C | 4.30E-08 | -- | 1.17E-07 | 1.13E-01 |
| rs8106260 | 19 | 31931409 | C | G | 3.85E-08 | ++ | 1.03E-07 | 1.19E-01 |
| rs6510206 | 19 | 31931845 | T | C | 4.54E-08 | -- | 1.24E-07 | 1.12E-01 |
| rs1074607 | 19 | 31932602 | A | G | 3.82E-08 | -- | 1.04E-07 | 1.14E-01 |
| rs4804966 | 19 | 31933275 | A | G | 3.51E-08 | -- | 9.50E-08 | 1.16E-01 |
| rs435991 | 19 | 31936411 | C | G | 3.84E-08 | -- | 1.01E-07 | 1.23E-01 |
| rs4812281 | 20 | 59794157 | T | C | 3.34E-08 | ++ | 1.01E-07 | 8.89E-02 |
| rs713843 | 22 | 27258265 | A | C | 4.52E-08 | ++ | 2.12E-07 | 2.55E-02 |
| rs8140423 | 22 | 38195596 | A | G | 3.64E-08 | -- | 7.86E-08 | 1.91E-01 |
| rs932376 | 22 | 42614362 | T | C | 1.88E-08 | -- | 6.49E-08 | 6.59E-02 |
| rs5751245 | 22 | 42620940 | T | C | 1.37E-08 | -- | 6.17E-08 | 3.30E-02 |

*chr: chromosome; p.Meta: meta-analysis p-value; p.Davies: p-value in the Davies et al (2018) study; p.SOL: p-value in the present study.*

**Supplementary Table 7. Functional annotation of variants associated with cognitive function. All candidate variants in associated genomic loci with an r^2^≥0.6 with one of the index genome-wide significant SNPs (IndSigSNP) and a suggestive p-value (p<10^−5^) are shown.**

| Variant | chr | Position | gwasP | IndSigSNP | r^2^ | Nearest gene | dist | Location | CADD | RDB | Min./Com. Chrom State | eQTL_DB | eQTL_Gene |
| --- | --- | --- | --- | --- | --- | --- | --- | --- | --- | --- | --- | --- | --- |
| rs112178366 | 4 | 40433442 | **4.06E-08** | rs113719683 | 0.99 | *RBM47* | 0 | intronic | 2.1 | 7 | 4/15 | GTEx/v7 | *APBB2; RBM47; N4BP2; UBE2K* |
| rs113719683 | 4 | 40433446 | **3.70E-08** | rs113719683 | 1.00 | *RBM47* | 0 | intronic | 5.4 | 7 | 4/15 | GTEx/v7 | *APBB2; RBM47; N4BP2; UBE2K* |
| rs112927755 | 4 | 40433460 | **4.32E-08** | rs113719683 | 0.98 | *RBM47* | 0 | intronic | 3.9 | 6 | 4/15 | GTEx/v7 | *APBB2; RBM47; N4BP2; UBE2K* |
| rs150206471 | 10 | 16220142 | **7.44E-09** | rs74610382 | 0.95 | *RNU6-1075P* | 29 677 | intergenic | 1.0 | 7 | 5/15 |  |  |
| rs116623781 | 10 | 16226698 | **5.05E-09** | rs74610382 | 0.81 | *RNU6-1075P* | 23 121 | intergenic | 1.4 | 5 | 5/15 |  |  |
| rs74610382 | 10 | 16254360 | **5.04E-09** | rs74610382 | 1.00 | *RNU6-1075P* | 4433 | intergenic | 3.0 | 7 | 9/15 |  |  |
| rs181182078 | 10 | 16260622 | **5.94E-09** | rs74610382 | 1.00 | *RNU6-1075P* | 10 695 | intergenic | 1.3 | 7 | 5/15 |  |  |
| rs142289140 | 10 | 16264277 | **8.31E-09** | rs74610382 | 1.00 | *RNU6-1075P* | 14 350 | intergenic | 0.5 | 6 | 8/9 |  |  |
| rs115696258 | 10 | 16270189 | **1.11E-08** | rs74610382 | 0.95 | *RNU6-1075P* | 20 262 | intergenic | 0.6 | 7 | 5/15 |  |  |
| rs12633344 | 3 | 69585352 | 4.99E-06 | rs599112956 | 0.61 | *FRMD4B:RP11-62G11.1* | 0:0 | ncRNA_exonic | 3.9 | 7 | 4/14 | BIOSQTL, GTEx/v7 | *RBM43P1,FRMD4B* |
| rs12633390 | 3 | 69585552 | 3.11E-06 | rs599112956 | 0.61 | *FRMD4B:RP11-62G11.1* | 0:0 | ncRNA_exonic | 9.0 | 5 | 4/14 | GTEx/v7 | *RBM43P1,FRMD4B* |
| rs148776110 | 3 | 69587453 | 1.38E-07 | rs599112956 | 0.83 | *FRMD4B* | 0 | intronic | 6.4 |  | 5/14 | GTEx/v7 | *RBM43P1,FRMD4B* |
| rs12633054 | 3 | 69587666 | 1.21E-07 | rs599112956 | 0.83 | *FRMD4B* | 0 | intronic | 0.1 | 4 | 5/14 | GTEx/v7 | *RBM43P1,FRMD4B* |
| rs599112956 | 3 | 69592250 | **5.09E-10** | rs599112956 | 1.00 | *FRMD4B* | 515 | upstream | 0.6 |  | 1/13 |  |  |
| rs12629703 | 3 | 69597914 | 2.29E-06 | rs599112956 | 0.63 | *FRMD4B* | 6179 | intergenic | 1.2 | 7 | 5/15 | GTEx/v7 | *RBM43P1,FRMD4B* |
| rs12633166 | 3 | 69599093 | 1.70E-07 | rs599112956 | 0.67 | *FRMD4B* | 7358 | intergenic | 9.2 | 6 | 5/15 | GTEx/v7 | *RBM43P1,FRMD4B* |
| rs62250896 | 3 | 69601011 | 1.37E-06 | rs599112956 | 0.62 | *FRMD4B* | 9276 | intergenic | 3.7 | 5 | 5/15 | GTEx/v7 | *RBM43P1,FRMD4B* |
| rs568391433 | 6 | 33004530 | **1.07E-08** | rs568391433 | 1.00 | *HLA-DOA* | 27 140 | intergenic | 15.0 |  | 7/15 |  |  |
| rs148920438 | 6 | 33066965 | 3.35E-06 | rs568391433 | 1.00 | *HLA-DPA2* | 1892 | intergenic | 1.2 | 7 | 8/14 |  |  |
| rs376243370 | 6 | 33210568 | 1.83E-06 | rs568391433 | 1.00 | *HTATSF1P* | 3100 | intergenic | 0.0 | 7 | 5/14 |  |  |
| rs367573391 | 6 | 33213669 | 1.81E-06 | rs568391433 | 1.00 | *XXbac-BPG157A10.21* | 182 | downstream | 5.7 | 7 | 5/14 |  |  |
| rs185251182 | 6 | 33220641 | 9.24E-07 | rs568391433 | 1.00 | *HCG25:VPS52* | 0:0 | ncRNA_intronic | **12.7** | 6 | 4/5 |  |  |
| rs190670955 | 6 | 33333198 | 9.39E-06 | rs568391433 | 0.80 | *LYPLA2P1* | 126 | downstream | **15.6** | 3a | 2/15 |  |  |
| rs192161187 | 6 | 33373544 | 9.66E-06 | rs568391433 | 0.80 | *KIFC1* | 0 | intronic | 0.1 | **2b** | 3/4 |  |  |

*chr: chromosome; gwasP: p-value of association in the GWAS analyses; IndSigSNP: Index SNP; dist: distance from nearest gene; CADD: CADD score; RDB: RegulomeDB score; Min./Com. Chrom State: minimum and most common 15-core chromatin state across 127 tissue/cell types. eQTL_DB: data source of eQTLs, including GTEx, BloodeQTL, BIOS and BRAINEAC; eQTL_Gene: gene whose expression is associated with corresponding SNP at FDR<0.05.*

**Supplementary Table 8. Top five genes for each cognitive test in gene-based analysis.**

| Trait | Gene | chr | No. variants | P-value |
| --- | --- | --- | --- | --- |
| SIS | *GPS2* | 17 | 198 | 1.17E-04 |
|  | *EIF5A* | 17 | 195 | 1.22E-04 |
|  | *NEURL4* | 17 | 232 | 1.50E-04 |
|  | *YBX2* | 17 | 165 | 1.62E-04 |
|  | *SLC2A4* | 17 | 159 | 1.84E-04 |
| B-SEVLT | *NFX1* | 9 | 483 | 1.13E-04 |
|  | *TEX101* | 19 | 611 | 1.24E-04 |
|  | *GRIA2* | 4 | 534 | 1.25E-04 |
|  | *BAG1* | 9 | 304 | 1.65E-04 |
|  | *CHMP5* | 9 | 293 | 1.80E-04 |
| WFT | *LINC00433* | 13 | 270 | 3.98E-05 |
|  | *NPTN* | 15 | 366 | 1.65E-04 |
|  | *SRSF5* | 14 | 344 | 1.95E-04 |
|  | *LOC100289511* | 14 | 321 | 2.06E-04 |
|  | *MLXIPL* | 7 | 436 | 2.82E-04 |
| DSST | *MDM4* | 1 | 391 | 4.26E-05 |
|  | *CPS1-IT1* | 2 | 300 | 4.76E-05 |
|  | *ABHD1* | 2 | 388 | 6.81E-05 |
|  | *PREB* | 2 | 381 | 7.28E-05 |
|  | *PRR30* | 2 | 377 | 7.75E-05 |

*chr: chromosome; SIS: Six-Item Screener; B-SEVLT: Brief Spanish English Verbal Learning Test; WFT: Word Fluency Test; DSST: Digit Symbol Substitution Test.*

**Supplementary Table 9. Polygenic risk score analyses using previous GWAS.**

| GWAS | N | Trait | Threshold (p-value) | No. variants | R^2^ | Empirical.P* |
| --- | --- | --- | --- | --- | --- | --- |
| Educational attainment | 766 345 | SIS | 1.50E-04 | 5524 | 0.19% | 2.10E-03 |
|  |  | B-SEVLT | 9.00E-04 | 10 788 | 0.32% | 2.00E-04 |
|  |  | WFT | 1.00E-04 | 4830 | 0.63% | <1.00E-04 |
|  |  | DSST | 6.00E-04 | 9192 | 0.49% | <1.00E-04 |
|  |  | G | 6.00E-04 | 9192 | 0.83% | <1.00E-04 |
| General cognitive function | 257 828 | SIS | 5.00E-04 | 5210 | 0.44% | <1.00E-04 |
|  |  | B-SEVLT | 6.50E-04 | 5887 | 0.45% | <1.00E-04 |
|  |  | WFT | 5.00E-04 | 5210 | 0.73% | <1.00E-04 |
|  |  | DSST | 4.00E-04 | 4665 | 0.85% | <1.00E-04 |
|  |  | G | 4.00E-04 | 4665 | 1.10% | <1.00E-04 |
| Reaction time | 330 069 | SIS | 4.43E-02 | 107 694 | 0.04% | 0.23 |
|  |  | B-SEVLT | 2.00E-04 | 2002 | 0.01% | 0.96 |
|  |  | WFT | 7.05E-03 | 25 179 | 0.01% | 0.98 |
|  |  | DSST | 1.00E-04 | 1302 | 0.02% | 0.94 |
|  |  | G | 7.05E-03 | 25 179 | 0.03% | 0.94 |
| Major depression disorder | 173 005 | SIS | 5.00E-01 | 481 498 | 0.04% | 0.38 |
|  |  | B-SEVLT | 3.20E-03 | 8300 | 0.04% | 0.87 |
|  |  | WFT | 2.05E-03 | 5758 | 0.03% | 0.97 |
|  |  | DSST | 9.25E-03 | 20 063 | 0.02% | 1 |
|  |  | G | 2.20E-03 | 6080 | 0.02% | 1 |
| Neuroticism | 168 105 | SIS | 1.94E-02 | 29 266 | 0.12% | 2.39E-02 |
|  |  | B-SEVLT | 3.00E-04 | 1303 | 0.03% | 0.88 |
|  |  | WFT | 2.50E-01 | 212 652 | 0.09% | 0.26 |
|  |  | DSST | 1.76E-01 | 163 953 | 0.07% | 0.52 |
|  |  | G | 1.76E-01 | 163 953 | 0.11% | 0.23 |
| Schizophrenia | 80 079 | SIS | 1.04E-02 | 21 070 | 0.06% | 0.11 |
|  |  | B-SEVLT | 1.50E-04 | 2039 | 0.07% | 0.25 |
|  |  | WFT | 1.50E-04 | 2039 | 0.01% | 1 |
|  |  | DSST | 1.00E-04 | 1667 | 0.10% | 0.16 |
|  |  | G | 1.50E-04 | 2039 | 0.07% | 0.51 |
| Alzheimer's disease | 54 162 | SIS | 1.45E-03 | 2213 | 0.10% | 4.66E-02 |
|  |  | B-SEVLT | 2.85E-03 | 3663 | 0.08% | 0.26 |
|  |  | WFT | 8.30E-02 | 52 712 | 0.01% | 1 |
|  |  | DSST | 2.00E-04 | 530 | 0.11% | 0.16 |
|  |  | G | 2.85E-03 | 3663 | 0.09% | 0.33 |
| White matter hyperintensities | 21 079 | SIS | 8.50E-04 | 1883 | 0.03% | 0.80 |
|  |  | B-SEVLT | 2.00E-04 | 540 | 0.13% | 0.10 |
|  |  | WFT | 2.00E-04 | 540 | 0.04% | 0.97 |
|  |  | DSST | 2.40E-03 | 4599 | 0.05% | 0.97 |
|  |  | G | 2.00E-04 | 540 | 0.09% | 0.61 |
| Hippocampal volume | 9232 | SIS | 6.00E-04 | 410 | 0.04% | 0.64 |
|  |  | B-SEVLT | 5.00E-04 | 357 | 0.01% | 1 |
|  |  | WFT | 3.48E-02 | 13 192 | 0.04% | 0.97 |
|  |  | DSST | 3.50E-03 | 1798 | 0.08% | 0.62 |
|  |  | G | 3.25E-03 | 1671 | 0.03% | 1 |

**Empirical p-value that accounts for multiple testing and over-fitting. GWAS: genome-wide association study; N: sample size; R^2^: proportion of phenotypic variance explained by polygenic risk score; SIS: Six-Item Screener; B-SEVLT: Brief Spanish English Verbal Learning Test; WFT: Word Fluency Test; DSST: Digit Symbol Substitution Test; G: general cognitive function (PC1).*

**Supplementary Figures**

**Supplementary Figure 1.** Quantile-Quantile plots for the genome-wide association study of **(A)** Six-Item Screener (SIS); **(B)** Brief Spanish English Verbal Learning Test (B-SEVLT); **(C)** Word Fluency Test (WFT); **(D)** Digit Symbol Substitution Test (DSST); **(E)** and general cognitive function (G).

**(B)**

**(A)**

**
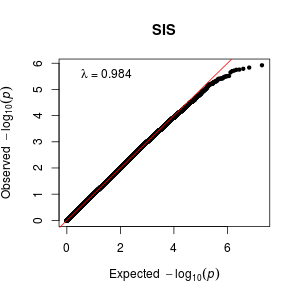

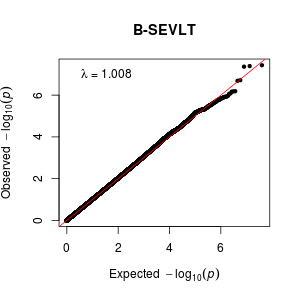

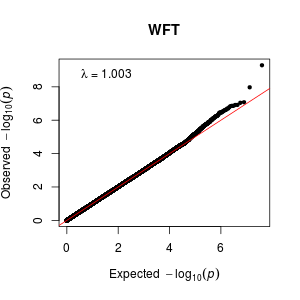

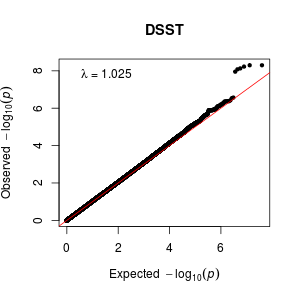
**

**(D)**

**(C)**

**(E)**


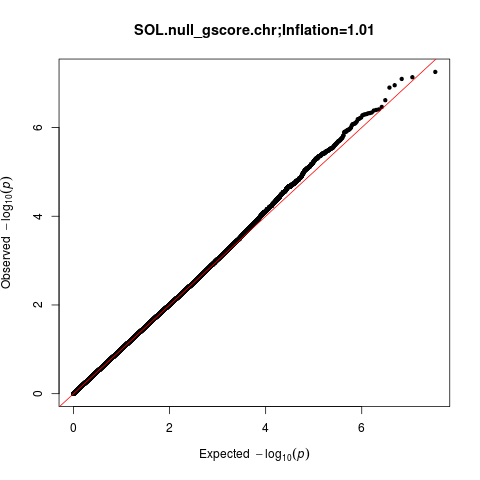


**G**

λ=1.01

**Supplementary Figure 2.** Manhattan plots for the meta-analysis of the general cognitive function in the present study with that in the Davies et al (2018) study, restricting to over 20 000 suggestive variants (10^−5^<*p*<5×10^−8^) from that study. The red horizontal line represents the genome-wide significance threshold (*p*<5×10^−8^). The green dot represents the significant variants in the meta-analysis that are independent of the genome-wide signifcant loci in Davies et al (2018) (r^2^<0.6).


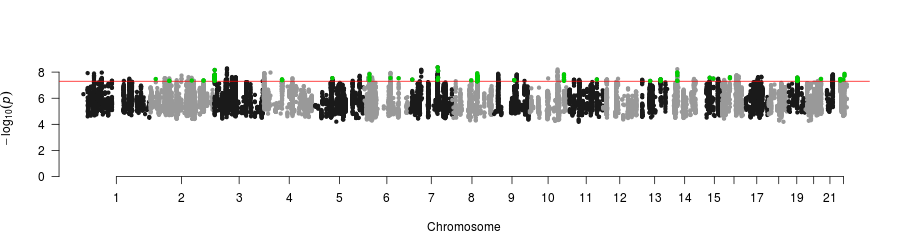


**Supplementary Figure 3.** Fine-mapping of the genome-wide significant locus at 10p13 for the Digit Symbol Substitution Test (DSST). The upper part plots the variants at this locus in terms of the p-values from the genome-wide association study (GWAS). The lower part plots the same set of variants in terms of the posterior probability of association (PPA) using RiVIERA. The red dot represents the lead GWAS variant, which also has the largest PPA. The green dots represent the 95% credible set containing the minimal number of variants whose PPA sum to 0. 95 or greater.

**
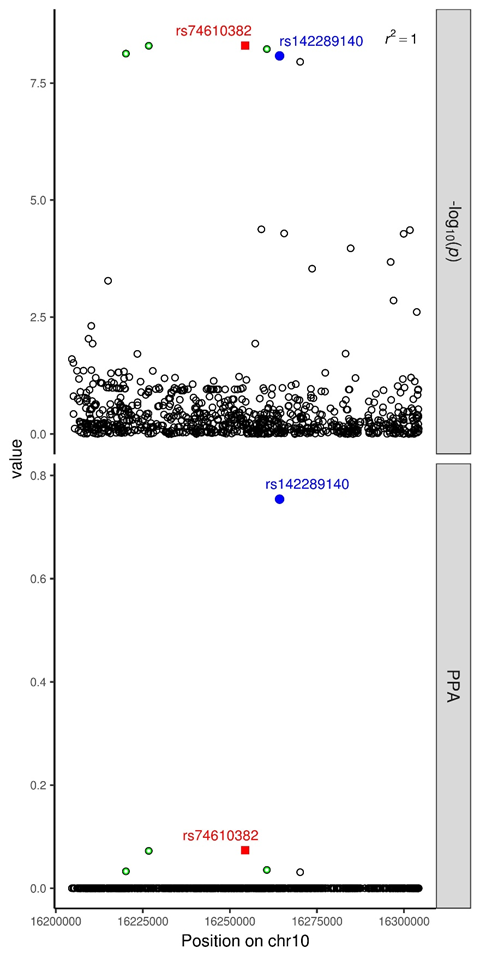
**

**Supplementary Figure 4.** Manhattan plots for the association of predicted genetically regulated gene expression in the **(A)** hippocampus for Six-Item Screener (SIS); **(B)** basal ganglia for Brief Spanish English Verbal Learning Test (B-SEVLT); **(C)** frontal cortex for Word Fluency Test (WFT); and **(D)** hypothalamus for Digit Symbol Substitution Test (DSST). The red horizontal line represents the Bonferroni-corrected significance threshold. The green dot represents the top gene in each analysis.

**(A)**


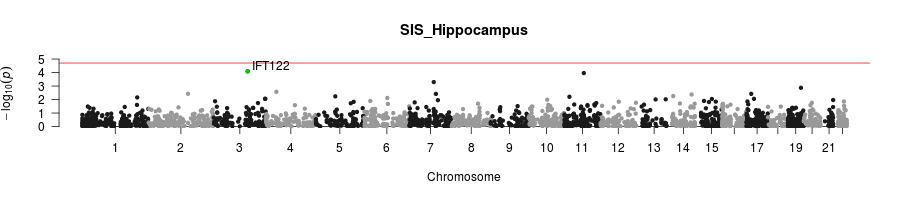
**
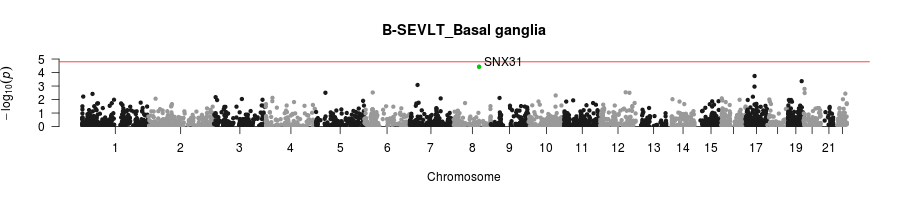
**
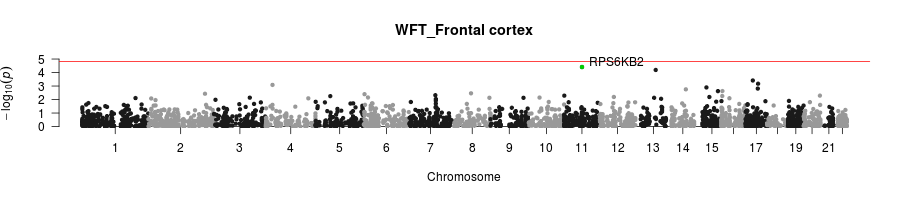

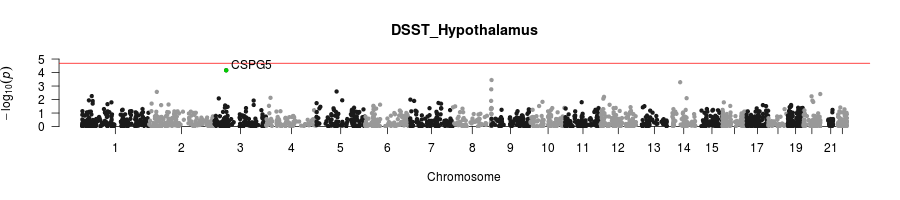


**(D)**

**(C)**

**(B)**

**Supplementary Figure 5.** Regional plots for the association of single variants within the genes suggested in PrediXcan, including **(A)** *IFT122* for Six-Item Screener (SIS); **(B)** *SNX31* for Brief Spanish English Verbal Learning Test (B-SEVLT); **(C)** *RPS6KB2* for Word Fluency Test (WFT); and **(D)** *CSPG5* for Digit Symbol Substitution Test (DSST).

**(B)**

**(A)**


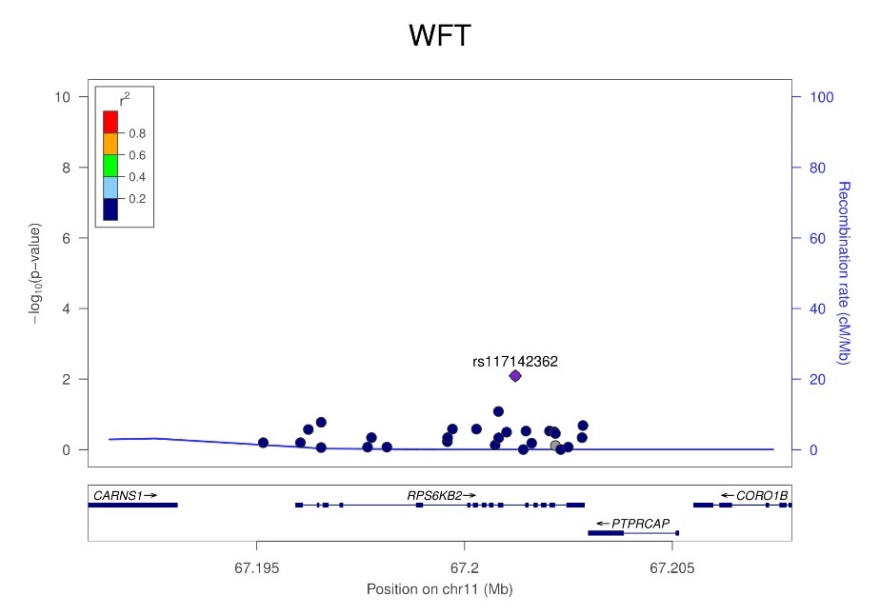

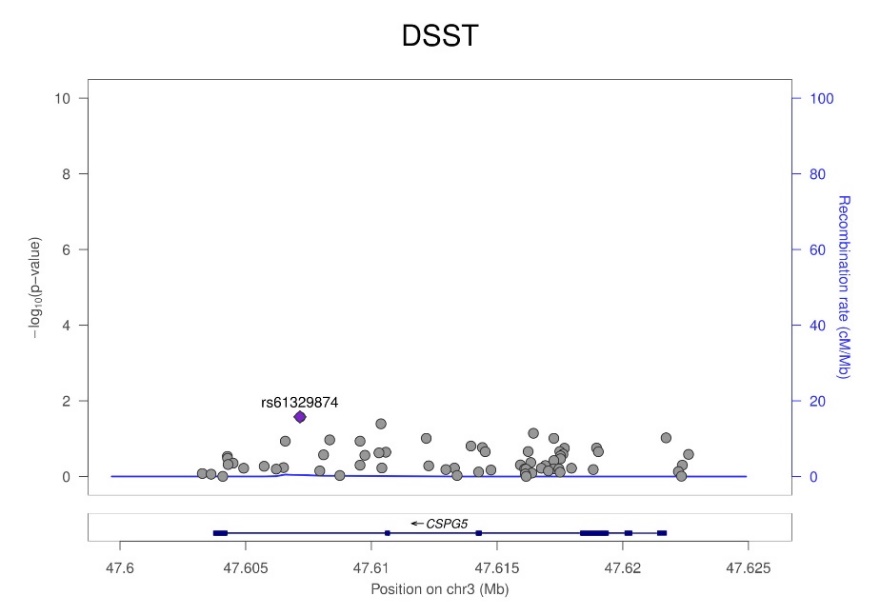

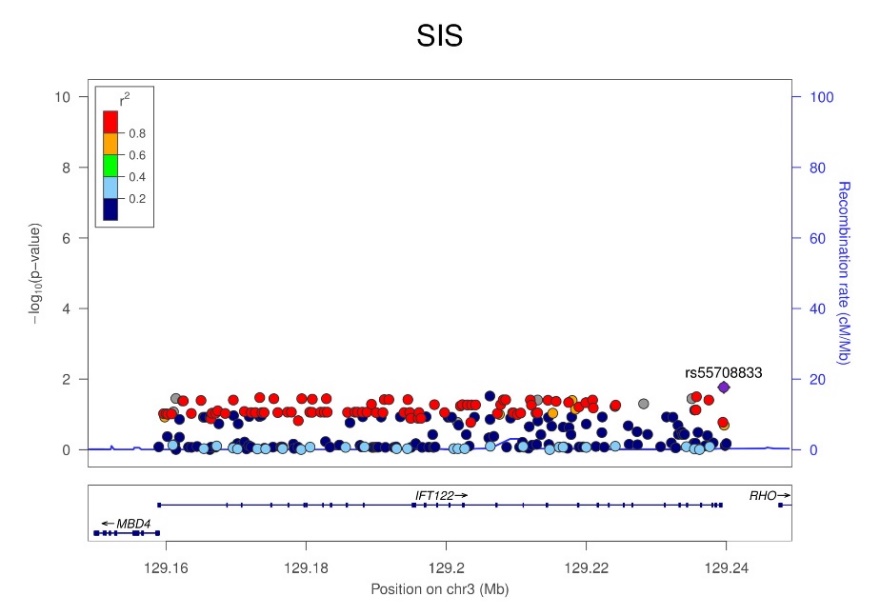

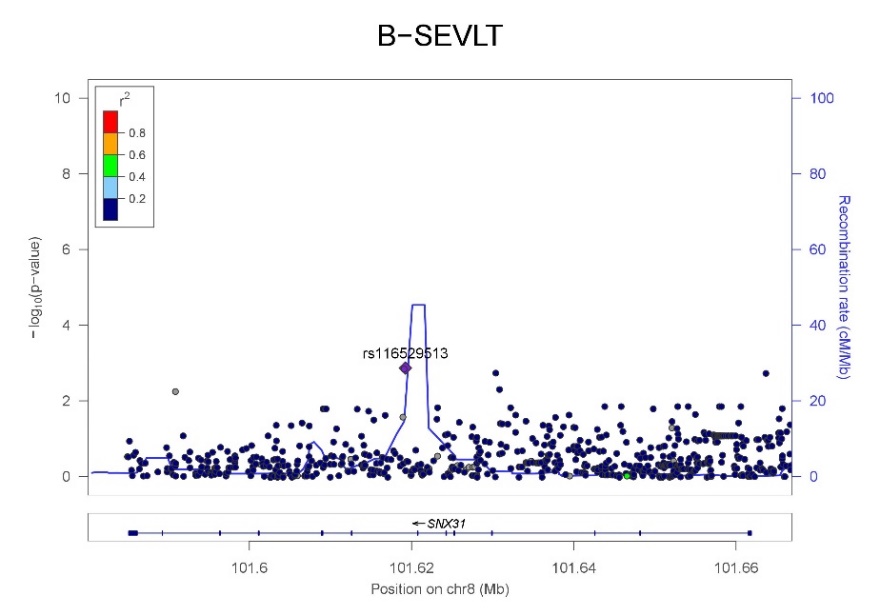


**(D)**

**(C)**

**
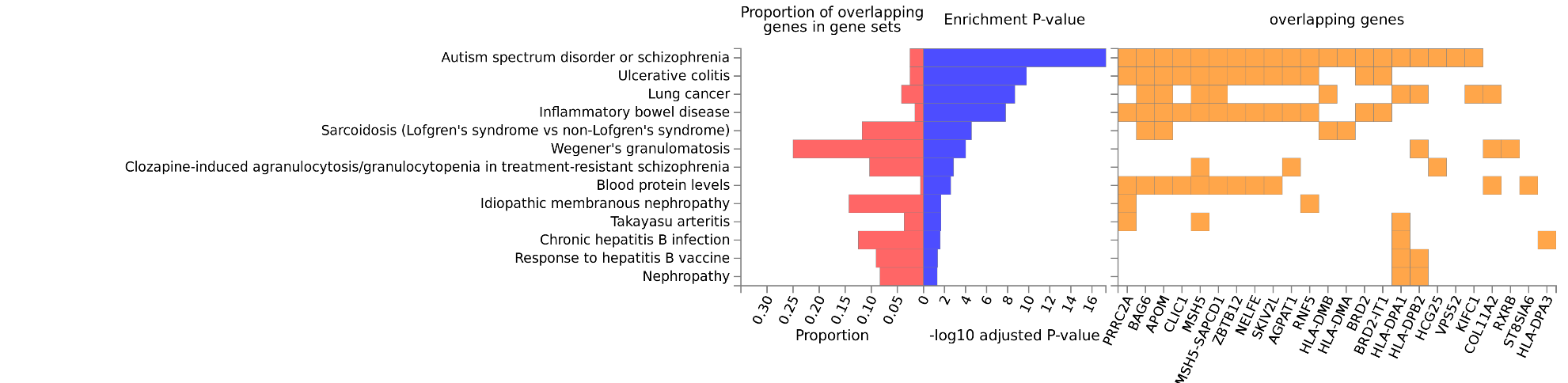
Supplementary Figure 6.** Gene set enrichment analysis of the 50 genes linked to the 4 cognitive function-associated loci by positional, eQTL, and chromatin-interaction mapping using the GENE2FUNC procedure in FUMA. The implicated genes were tested for over-representation among 2195 GWAS catalog gene-sets. There was a significant enrichment for genes associated with autism and schizophrenia.
